# Orthogonal ubiquitin transfer identifies ubiquitination substrates under differential control by the two ubiquitin activating enzymes

**DOI:** 10.1038/ncomms14286

**Published:** 2017-01-30

**Authors:** Xianpeng Liu, Bo Zhao, Limin Sun, Karan Bhuripanyo, Yiyang Wang, Yingtao Bi, Ramana V. Davuluri, Duc M. Duong, Dhaval Nanavati, Jun Yin, Hiroaki Kiyokawa

**Affiliations:** 1 Department of Pharmacology, Northwestern University, Chicago, Illinois 60611, USA; 2 Department of Chemistry, University of Chicago, Chicago, Illinois 60637, USA; 3 School of Pharmacy, Shanghai Jiao Tong University, Shanghai 20040, China; 4 Department of Chemistry, Center for Diagnostics & Therapeutics, Georgia State University, Atlanta, Georgia 30303, USA; 5 Department of Preventive Medicine, Northwestern University, Chicago, Illinois 60611, USA; 6 Robert H. Lurie Comprehensive Cancer Center, Northwestern University, Chicago, Illinois 60611, USA; 7 Integrated Proteomics Core, Emory University, Atlanta, Georgia 30322, USA; 8 Chemistry of Life Processes Institute, Northwestern University, Chicago, Illinois 60611, USA

## Abstract

Protein ubiquitination is mediated sequentially by ubiquitin activating enzyme E1, ubiquitin conjugating enzyme E2 and ubiquitin ligase E3. Uba1 was thought to be the only E1 until the recent identification of Uba6. To differentiate the biological functions of Uba1 and Uba6, we applied an orthogonal ubiquitin transfer (OUT) technology to profile their ubiquitination targets in mammalian cells. By expressing pairs of an engineered ubiquitin and engineered Uba1 or Uba6 that were generated for exclusive interactions, we identified 697 potential Uba6 targets and 527 potential Uba1 targets with 258 overlaps. Bioinformatics analysis reveals substantial differences in pathways involving Uba1- and Uba6-specific targets. We demonstrate that polyubiquitination and proteasomal degradation of ezrin and CUGBP1 require Uba6, but not Uba1, and that Uba6 is involved in the control of ezrin localization and epithelial morphogenesis. These data suggest that distinctive substrate pools exist for Uba1 and Uba6 that reflect non-redundant biological roles for Uba6.

## Abstract (web summary)

The transfer of ubiquitin (UB) to cellular targets is mediated sequentially by three groups of enzymes, UB activating enzyme (E1), UB conjugating enzyme (E2) and UB ligase (E3). Here the authors provide evidence that the two mammalian E1 enzymes, Uba1 and Uba6, exert biologically distinct functions.

Ubiquitination, covalent conjugation of cellular proteins with the small regulatory protein ubiquitin (UB), plays diverse roles in controlling the fate of the substrates, such as proteolysis, altered subcellular localization and modulated enzymatic activities^1^. UB transfer to cellular targets is mediated sequentially by three groups of enzymes, UB activating enzyme (E1), UB conjugating enzyme (E2) and UB ligase (E3) (ref. 2). UB is first activated by E1, which consumes ATP to form a thioester bond between the active site cysteine and the carboxyl terminus of UB (ref. 3). Subsequently, E1 engages one of ∼30 E2s to bridge UB transfer to substrate proteins recruited by E3s (refs 4, 5). Human genome contains two E1 genes for ubiquitination, *UBA1* and *UBA6*. Historically, Uba1 protein, also known as Ube1, was designated as the sole E1 for all the ubiquitination reactions, since mammalian cells harbouring temperature-sensitive mutations in the *Uba1* gene demonstrated rapid loss of ubiquitin-activating capacity, stabilization of short-lived proteins and G_2_ cell cycle arrest at non-permissive temperatures^6^,^7^,^8^,^9^. However, this notion was challenged by the recent identification of Uba6 as an alternative or non-canonical E1 for UB activation^10^,^11^,^12^. Uba1 and Uba6 are expressed ubiquitously, and it is thought that they are interchangeable in many ubiquitination events by transferring UB to a shared pool of E2s and E3s. Indeed, several E2 enzymes such as UBE2D1-4/UbcH5a-d, UBE2G2, UBE2L3/UbcH7, UBE2S and UBE2T have been shown *in vitro* to uptake UB from both Uba1 and Uba6. In contrast, UBE2Z/Use1 is recognized exclusively by Uba6 for UB loading^11^. The E1-E2 pair composed of Uba6 and UBE2Z transfers not only UB but also the UB-like modifier FAT10 (ref. 13). Embryonic lethality of *Uba6*-null mice suggests an indispensable role for Uba6 in development^10^,^14^. Mice with brain-specific *Uba6* disruption exhibit abnormal patterning of neurons with a phenotype similar to autism spectrum diseases, implying the importance of Uba6 in neuronal development and function^14^. On the other hand, Mice with *Fat10* disruption are viable with modest metabolic changes^15^,^16^, underlining the developmental significance of Uba6-dependent ubiquitination. So far only a few proteins have been described to be ubiquitinated in Uba6-dependent manners, such as RGS4, RGS5, UBE3A/E6-AP and Shank3 (refs 14, 17), and the ubiquitination pathways initiated by Uba6 remain obscure. To better understand the non-canonical activity of Uba6 in ubiquitination, here we applied a novel technique named Orthogonal Ubiquitin Transfer (OUT) (ref. 18) to differentiate the cellular ubiquitination targets of Uba1 and Uba6. We engineered UB so that the UB mutant (xUB) could not be activated by the wild-type (wt) Uba1 or Uba6. Correspondingly we engineered the UB binding sites in Uba1 and Uba6 to restore their activities with xUB while eliminating their activities with wt UB. In this way the xUB-xUba1 and xUB-xUba6 pairs would transfer xUB through either xUba1 or xUba6 to their partner E2 and E3 enzymes and further to ubiquitination targets. By expressing the xUB-xUba1 pair and xUB-xUba6 pair separately in mammalian cells, we identified partially overlapping yet distinctive pools of cellular proteins that are potential targets of Uba1 or Uba6 initiated ubiquitination.

## Results

### Generating the orthogonal pairs of xUB-xUBA6 and xUB-xUBA1

**Figure 1:**
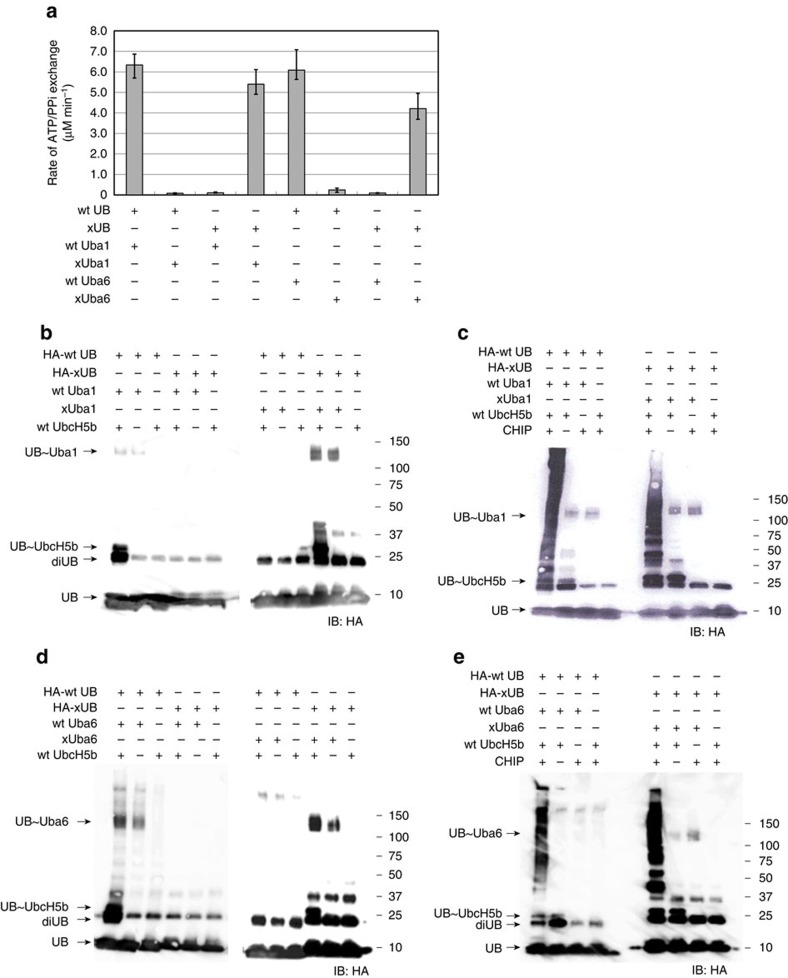
*In vitro* orthogonal reactivity of the xUB-xUba6 and xUB-xUba1 pairs. (**a**) xUB and wt UB activation by the xE1 and wt E1 enzymes in the ATP-PPi exchange assay. Data are shown as means±s.e.m. (*n*=3). (**b**) Formation of UB∼E1 and UB∼E2 thioester conjugates with xUB and xUba1. UBE2D2/UbcH5b was used as the E2. Cross-reactivity between xUB and wtUba1, and between wtUB and xUba1 was not detected. (**c**) xUba1 can transfer xUB to UbcH5b and CHIP. CHIP autoubiquitination by wt UB and wt Uba1 were used as a positive control. (**d**) Same as (**b**) with the xUB-xUba6 pair. (**e**) Same as (**c**) with the xUB-xUba6 pair.

We previously used phage display to engineer an xUB-xUba1 pair with Uba1 from *Saccharomyces cerevisiae* to enable the activation of xUB by xUba1 (ref. 18). Mutations R42E and R72E were incorporated into xUB to block its recognition by wt Uba1. Subsequently, mutations Q576R, S589R and D591R were introduced into the adenylation domain of xUba1 to complimentarily restore its interaction with xUB (Supplementary Fig. 1a,b). xUB activation by xUba1 was approaching the efficiency of wt UB activation by wt Uba1, whereas xUB activation by wt Uba1 or wt UB activation by xUba1 were almost 1,500-fold lower than the wt UB-Uba1 pair or the engineered xUB-xUba1 pair^18^. Analysis of the crystal structures of Uba1 from *S. cerevisiae* and *Schizosaccharomyces pombe* in complex with UB reveals that R42 and R72 of UB are engaged in a network of hydrogen bonding and salt bridge interactions with Uba1 residues Q576, S589 and D591 (*S. cerevisiae*)^19^,^20^. A sequence comparison of the E1 enzymes from *S. cerevisiae, S. pombe* and human suggests a highly conserved UB binding pocket in the adenylation domain (Supplementary Fig. 1c). We thus rationalized that the corresponding residues in human Uba1 (Q608, S621 and D623) and Uba6 (E601, H614 and D616) would engage R42 and R72 in UB. Accordingly we generated corresponding mutants of human Uba1 (Q608R, S621R and D623R) as xUba1, and human Uba6 (E601R, H614R and D616R) as xUba6, and assayed their activities with xUB and wt UB. ATP-PPi exchange assay^21^,^22^ suggested that the xUB-xUba1 and xUB-xUba6 pairs had a similar rate of xUB activation as the wt UB-Uba1 and wt UB-Uba6 pairs (Fig. 1a). In contrast, the cross-reactivities of xUB with wt Uba1 and Uba6 were minimal. We also reacted xUB and wt UB with the wt and engineered human E1s. Western blots of the UB transfer reactions indicated that xUB could be transferred from either xUba1 or xUba6 to wt UBE2D2/UbcH5b. In contrast xUB was incapable of loading to wt Uba1 or Uba6 and further transfer to UbcH5b (Fig. 1b,d). Furthermore, once xUB was activated by either xUba1 or xUba6, they could be transferred to CHIP E3 (ref. 23) through UbcH5b to support CHIP auto ubiquitination (Fig. 1c,e). These results suggest that the xUB-xUba1 and xUB-xUba6 pairs are orthogonal to the wt UB-E1 pairs.

### Orthogonal interaction of xUB-xE1 in mammalian cells

**Figure 2:**
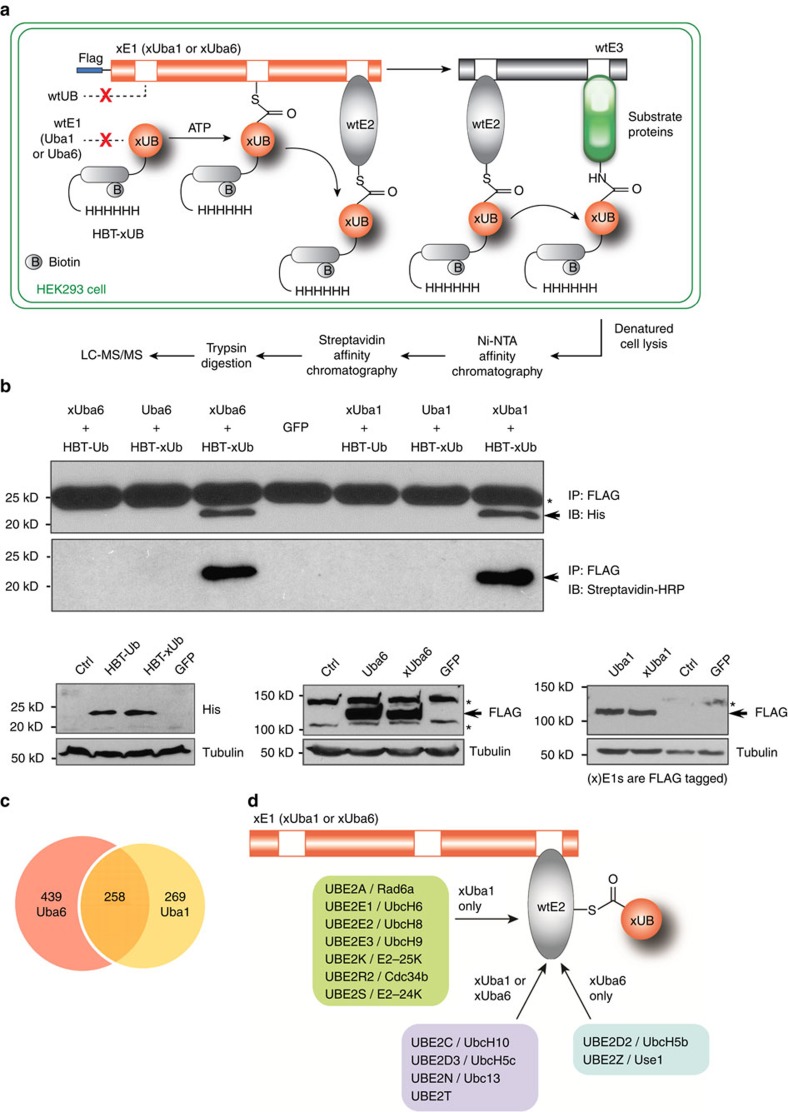
Profiling cellular substrates of Uba6- and Uba1-dependent ubiquitination. (**a**) Flow chart of procedures to identify xUba6- and xUba1-dependent ubiquitination substrates by tandem affinity purification and proteomic procedures. (**b**) Specific reactions of the xUB-xUba1 pair and the xUB-xUba6 pair in HEK293 cells with lentiviral transduction. Upper panels indicate that HBT(histidine/biotinylation-signal tag)-xUB physically conjugates with FLAG-xUba6 and FLAG-xUba1, whereas HBT-wt UB shows no conjugation with FLAG-xUba6 or FLAG-xUba1. HEK293 cells were infected with recombinant lentiviruses for expression of the indicated proteins, followed by drug selection for stable integration. Conjugation of wt UB or xUB with wt E1 or xE1 proteins was examined by immunoprecipitation for E1 proteins under a non-reducing condition, followed by immunoblotting for the poly-histidine tag of UB (the upper panel) or for the biotinylation tag with streptavidin conjugated with horse radish peroxidase (HRP) (the middle panel). The arrow indicates UB proteins, while the asterisk shows a common protein around 25 kDa with cross-reactivity for the anti-penta-histidine antibody. The bottom panels demonstrate total expression levels of each protein determined by direct immunoblotting for the indicated epitope tag or tubulin as a loading control. Ctrl, parental HEK293 cells without viral transduction; GFP, cells infected with a lentivirus for green fluorescent protein. (**c**) Numbers of Uba6-specific, Uba1-specific and Uba6/Uba1-shared ubiquitination substrates identified by the OUT screen. (**d**) E2 enzymes conjugated with HBT-xUB in xUba6- or xUba1-dependent manners. The enzymes shown in the centre were identified by both xUba6- and xUba1-mediated screens.

The orthogonal xUB-xUba1 and xUB-xUba6 pairs provided a platform to track xUB transfer and identify cellular ubiquitination targets in xUba6- or xUba1-dependent manners, as shown in Fig. 2a. To efficiently purify UB-conjugated proteins under denaturing conditions, we constructed lentiviral vectors to express xUB and wt UB with tandem poly-histidine-biotinylation signal tag (HBT). Previously, Kaiser *et al*. expressed HBT-wt UB in HeLa cells and identified 669 ubiquitinated proteins^24^, which demonstrated efficacy of the purification system. We then conducted sequential lentiviral infection in HEK293 cells to generate stable cell populations expressing pairs of HBT-xUB or HBT-wt UB with FLAG-xUba1, FLAG-wt Uba1, FLAG-xUba6 or FLAG-wt Uba6. Immunoblotting showed that all wt and mutant forms of UBA1 and UBA6 were expressed properly (Fig. 2b). Expression levels of HBT-xUB and HBT-wt UB were less than 10% of cellular endogenous UB (Supplementary Fig. 2). FLAG-tagged E1 enzymes were immunoprecipitated under non-reducing conditions, and associated HBT-UB proteins were detected by immunoblotting using anti-penta-histidine antibody or by probing with streptavidin conjugated with horse radish peroxidase. We found HBT-xUB co-immunoprecipitated efficiently with the E1-targeting anti-FLAG antibody from cells expressing the xUB-xUba1 or xUB-xUba6 pairs. In contrast, no HBT-xUB association was detected in cells expressing the crossing-over xUB-wt Uba1 or xUB-wtUba6 pairs. Neither HBT-wtUB association was detected in cells expressing wtUB-xUba1 or wtUB-xUba6 pairs. These results confirmed the orthogonality of xUB-xE1 pairs with the wt UB-E1 pairs in HEK293 cells.

### Identification of xUB-conjugated proteins

To purify HBT-xUB-conjugated cellular proteins, HEK293 cell populations expressing HBT-xUB+FLAG-xUba6 or HBT-xUB+FLAG-xUba1 were treated with the proteasome inhibitor MG132, and then lysed in a denaturing buffer. Lysates were subjected to tandem affinity chromatography with the Ni-NTA resin and streptavidin-agarose (Supplementary Fig. 3 for immunoblots at each purification step). Purified proteins were cleaved by trypsin and subjected to LC-MS/MS (Fig. 2a). As described in Methods, each protein identification was supported by peptide assignments with *q* value scores ≤ 0.1. To gain further confidence, any protein identification supported by single peptide assignments was manually validated. These procedures led to identifying 697 and 527 proteins conjugated with HBT-xUB from cells expressing xUba6 and cells expressing xUba1, respectively (Fig. 2c; see Supplementary Data 1–3 for detailed information of all identified proteins). Consistent with putative overlapping functions of Uba1 and Uba6 (ref. 11), 258 proteins were identified in both screens for xUba1- and xUba6-mediated ubiquitination and regarded as potential substrates shared by Uba1 and Uba6. Control screening with cells expressing HBT-xUB alone resulted in identification of 243 proteins, most of which are highly abundant proteins including cytoskeletal proteins, histones, energy metabolism enzymes, scaffolds and chaperones (Supplementary Data 4). These proteins were regarded as nonspecific background associated with tandem purification and removed from the data sets of xUba1- and xUba6-mediated ubiquitination targets. To further prioritize the hits among the filtered data sets, we used the CRAPome database, which consists of data from negative control experiments for affinity purification-mass spectrometry studies^25^. While CRAPome is based on non-denaturing affinity purification for interactome studies, most frequently detected proteins in the CRAPome database are highly abundant proteins that are likely to associate with resins in bait-independent manners^25^ and some of them might be detected nonspecifically even under denaturing conditions in the OUT screens. Of 439 Uba6-specific, 269 Uba1-specific and 258 Uba6/Uba1-shared targets, 25, 28 and 77 proteins, respectively, were found to be frequently detected proteins in CRAPome, and these proteins were categorized in groups with lower priority (grey-shaded in Supplementary Data 1–3). The Uba6-specific data set included two E2s (UBE2Z/Use1 and UBE2D2/UbcH5b), and the Uba1/Uba6-shared data set included four E2s (UBE2C/UbcH10, UBE2D3/UbcH5c, UBE2N/Ubc13 and UBE2T) (Fig. 2d). These E2s except UBE2N were previously demonstrated to uptake UB from Uba6 *in vitro* and UBE2N was not examined in the study^11^. The Uba1-specific data set included seven E2s (UBE2A/Rad6a, UBE2E1/UbcH6, UBE2E2/UbcH8, UBE2E3/UbcH9, UBE2K/E2-25k, UBE2R2/Cdc34b and UBE2S/E2-24k). Thus, these data confirmed the legitimate specificity of the OUT-based screens.

Bioinformatics analyses of the identified proteins by Ingenuity Pathway Analysis (IPA) showed that the three groups of cellular ubiquitination targets, that is, Uba6-specific, Uba1-specific and Uba6/Uba1-shared substrates, were associated with significantly different canonical pathways, while several pathways were associated with multiple groups of substrates (Supplementary Fig. 4, Supplementary Data 5). For example, 12 pathways including mitochondrial dysfunction, Cdc42 signalling and RhoA signalling showed statistically significant association with only Uba6-specific substrates (Supplementary Fig. 4, first group in the heat map). On the other hand, eight pathways including oxidative stress pathway and AMPK signalling were associated significantly with only Uba1-specific substrates (Supplementary Fig. 4, seventh group at the bottom of the heat map). IPA also provided protein networks from its database that were associated significantly with Uba6-specific, Uba1-specific and Uba6/Uba1-shared substrates, respectively (Supplementary Data 6).

### Validation of ezrin and CUGBP1 as Uba6-specific substrates

**Figure 3:**
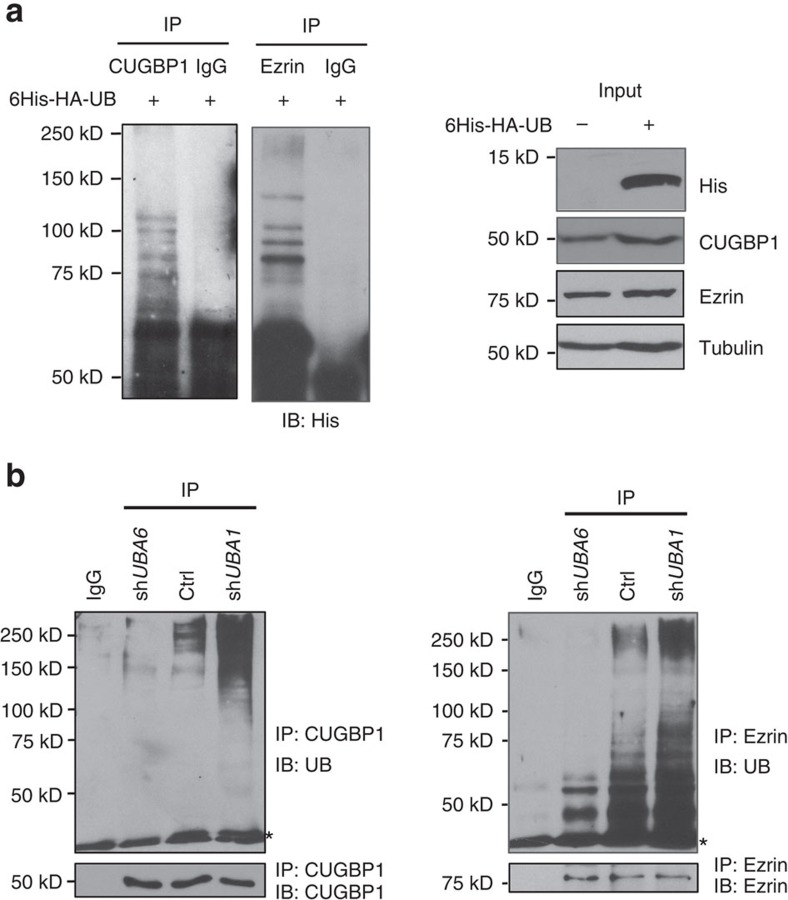
Uba6 is required for polyubiquitination of the RNA binding protein CUGBP1 and the actin-binding protein ezrin. (**a**) CUGBP1 and ezrin are polyubiquitinated. HEK293 cells were transfected with polyhistidine-HA-tagged ubiquitin (UB), and treated with 10 μM MG132 for 90 min. The indicated proteins were immunoprecipitated (IP), followed by immunoblotting (IB) for the polyhistidine tag. IgG, control immunoprecipitation with normal rabbit or mouse IgG. The right panels indicate immunoblotting with total cell lysates for the indicated proteins. (**b**) Silencing *UBA6* results in decreased polyubiquitination of CUGBP1 and ezrin, while silencing *UBA1* increases polyubiquitination of these proteins. HEK293 cells were lentivirally transduced with anti-*UBA6*, anti-*UBA1* or control (Ctrl) shRNA. Following drug selection, stable cell populations were immunoprecipitated followed by immunoblotting, as indicated. The asterisks indicate background bands from IgG.

**Figure 4:**
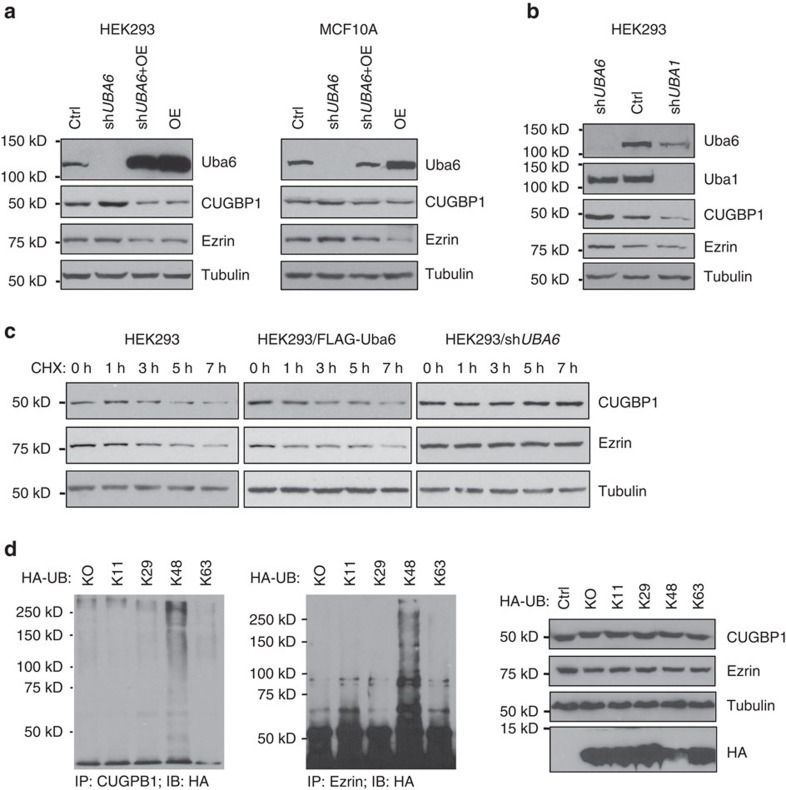
Uba6 negatively controls the stability of CUGBP1 and ezrin by mediating K48-linked polyubiquitination of the proteins. (**a**) Effects of *UBA6* silencing by shRNA (sh*UBA6*), overexpression (OE) of Uba6, or Uba6 overexpression in the sh*UBA6* background (sh*UBA6*+OE) upon the steady-state levels of the indicated proteins. HEK293 and MCF-10A cells were infected with recombinant lentiviruses to generate cell populations with Uba6 knockdown and/or overexpression, which were analysed by immunoblotting. The shRNA targets the 3′-untranslated region of the *UBA6* mRNA, and does not affect exogenous cDNA expression. (**b**) Effects of *UBA6* or *UBA1* silencing on the steady-state levels of the indicated proteins determined by immunoblotting. (**c**) Uba6 controls the degradation of CUGBP1 and ezrin. HEK293 cells were treated with 100 μg ml^−1^ cycloheximide for the indicated hours, and then examined by immunoblotting for CUGBP1 or ezrin to determine its half-life. (**d**) Polyubiquitiated forms of CUGBP1 and ezrin consist of K48-linked ubiquitin chains. HEK293 cells were transfected with hemagglutinin (HA)-tagged ubiquitin (UB) mutants that accept chain linkage only on the K11, K29, K48 or K63 residue, followed by immunoprecipitation (IP) of the target proteins and immunoblotting (IB) for the HA tag. The right panels show cellular levels of the indicated proteins determined by direct IB.

Since knowledge about the biological functions of Uba6 is limited, we conducted cell biological characterizations of representative Uba6-specific substrates. We chose the ezrin, radixin and moesin family actin binding protein ezrin^26^,^27^ and the RNA binding protein CUGBP1 (also known as CELF1) (ref. 28) and attempted to verify their authenticity as Uba6-specific ubiquitination substrates. These proteins were components of IPA networks that show highly significant association (Supplementary Data 6, ID 2 and 5). Cells were transfected with UB tagged with polyhistidine, treated with MG132, and harvested for immunoprecipitation with CUGBP1 or ezrin. Immunoblotting with polyhistidine antibody demonstrated that these proteins were polyubiquitinated in HEK293 cells (Fig. 3a). To examine the effects of *UBA6* or *UBA1* silencing, HEK293 cells were infected with recombinant lentivirus encoding anti-*UBA6* or anti-*UBA1* shRNA, drug selected, and analysed by immunoprecipitation with antibodies against each ubiquitination target followed by immunoblotting for endogenous UB. Polyubiquitinated forms of CUGBP1 and ezrin were significantly diminished in cells expressing anti-*UBA6* shRNA, relative to controls (Fig. 3b), which is consistent with the notion that ubiquitination of these proteins depends on Uba6. In contrast, cells expressing anti-*UBA1* shRNA exhibited enhanced polyubiquitination of CUGBP1 and ezrin. These data support the notion that CUGBP1 and ezrin are Uba6-specifi targets. Further analysis of the effects of Uba6 or Uba1 knockdown showed that polyubiquitination controlled the stability and steady-state levels of CUGBP1 and ezrin. Cellular levels of CUGBP1 and ezrin were significantly increased by anti-*UBA6* shRNA in HEK293 cells and human mammary epithelial MCF-10A cells (Fig. 4a). These increases were abolished by forced overexpression of FLAG-tagged Uba6 in cells stably expressing anti-*UBA6* shRNA, indicating that the observed actions of the shRNA did not result from any off-target effect. Overexpression of FLAG-Uba6 in the two cell lines resulted in significant decreases in ezrin levels and also in CUGBP1 levels to lesser extents. Similar effects of Uba6 knockdown on cellular levels of ezrin and CUGBP1 were observed in human prostate epithelial RWPE-1 cells and prostate carcinoma PC3M cells (Supplementary Fig. 5). Moreover, anti-*UBA1* shRNA decreased levels of CUGBP1 and ezrin in HEK293 cells (Fig. 4b). Experiments using the protein synthesis inhibitor cycloheximide demonstrated that overexpression of FLAG-Uba6 shortened the half-lives of CUGBP1 and ezrin, whereas silencing of *UBA6* stabilized these proteins (Fig. 4c). To assess polyubiquitin linkages on CUGBP1 and ezrin, HEK293 cells were transfected with HA-tagged UB mutants that accept chain linkage only on the K11, K29, K48 or K63 residue, followed by immunoprecipitation of the target proteins and immunoblotting for the HA tag (Fig. 4d). This study exhibited that polyubiquitinated forms of CUGBP1 and ezrin consisted predominantly of UB chains with the canonical K48 linkage, which is consistent with the effects of *UBA6* silencing on the stability of these proteins and also with a previous study that demonstrated K48-linked polyubiquitination of RGS proteins mediated by Uba6 and UBE2Z (ref. 17). Actually, not only *UBA6* silencing but also *UBE2Z* silencing could increase cellular steady-state levels of CUGBP1 and ezrin, suggesting the involvement of UBE2Z, a Uba6-specific E2, in K48-linked polyubiquitination of these newly defined substrates (Supplementary Fig. 6). Taken together, Uba6 mediates K48-linked polyubiquitination of CUGBP1 and ezrin to induce proteasomal degradation of these substrates. These data indicate that the two representative ubiquitination substrates, CUGBP1 and ezrin, identified by the xUB-xUba6 screen were bona fide Uba6-specific targets.

### Uba6 controls acini-like morphogenesis of epithelial cells

**Figure 5:**
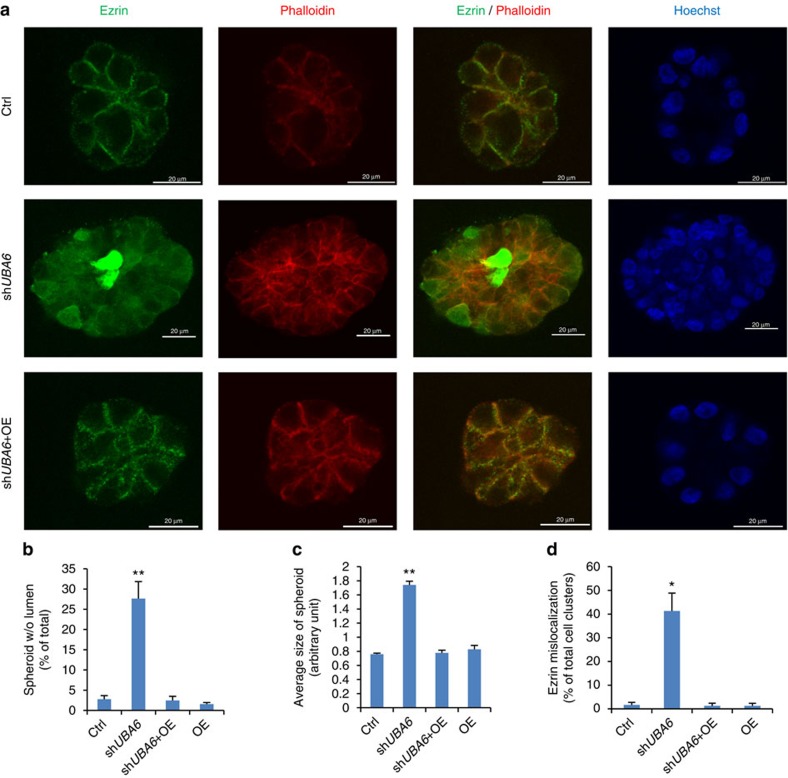
Uba6 is required for the regulation of subcellular localization of ezrin and formation of epithelial acini in human nontransformed mammary epithelial MCF-10A cells. (**a**) Immunofluorescence microscopy for ezrin, F-actin (via phalloidin) and chromosomal DNA (via Hoechst 33342) in cell spheroids formed in 3D culture at day 14. Representative pictures are shown from three groups such as control, sh*UBA6* and *shUBA6* plus FLAG-Uba6 cDNA (OE). (**b**) Quantification of spheroids without lumen in day 14 culture. (**c**) The average size of spheroids in each experimental group relative to the control group. The mean acinar structure area in each group was quantified using ImageJ software (see Supplementary Fig. 7). (**d**) Quantification of spheroids with mislocalized ezrin. **P<0.01; *P<0.05. Data are shown as means+s.e.m. from three biological replicates.

**Figure 6:**
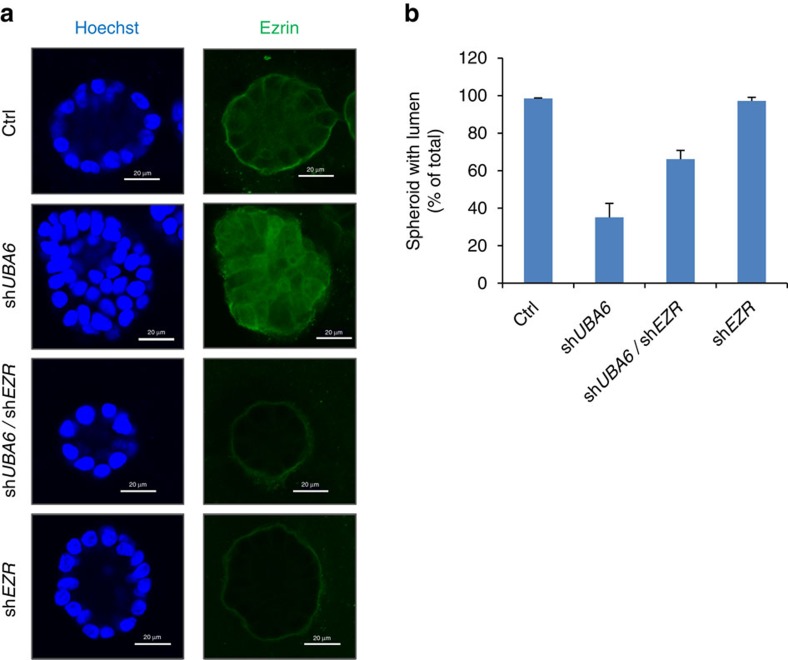
Silencing of ezrin partially rescues *UBA6-*deficient MCF-10A cells from perturbed epithelial morphogenesis in 3D culture. Human non-transformed mammary epithelial MCF-10A cells stably expressing the indicated shRNAs were generated by lentiviral transduction and drug selection, and examined for epithelial morphogenesis by 3D culture. (**a**) Immunofluorescence microscopy for ezrin and chromosomal DNA (via Hoechst 33342) in cell spheroids formed in 3D culture. (**b**) Quantification of spheroids with lumen formation. Data are shown as means+s.e.m. from three biological replicates.

Since ezrin controls apical-basal polarity during epithelial morphogenesis^29^, we examined whether Uba6-dependent ubiquitination controls acinar morphogenesis using mammary epithelial cells in three-dimensional (3D) culture. MCF-10A cells are non-transformed and undergo differentiation to form highly organized acini-like spherical structures when cultured on laminin-rich basement membrane such as Matrigel. This experimental system has been widely used to study roles of various genes in the formation of epithelial acini^30^,^31^. We generated MCF-10A cell populations that stably express anti-*UBA6* shRNA or control shRNA together with or without FLAG-Uba6 cDNA, by serial lentiviral transduction and drug selection. Cells were then cultured in chambers with medium containing Matrigel for the 3D environment. Control cells cultured in 3D for 14 days exhibited acinar formation characteristic of ductal morphogenesis, as expected, and >90% of spheroids showed internal lumen formation as observed by confocal microscopy (Fig. 5a,b). In contrast, MCF-10A cells with stable Uba6 knockdown exhibited significantly larger epithelial acini, ∼30% of which lacked the typical lumen formation (Fig. 5b,c). Immunofluorescence microscopy further demonstrated that ezrin expression in control cells was enriched predominantly at the plasma membrane (Fig. 5a, Supplementary Fig. 7). In contrast, ezrin expression in cells expressing anti-*UBA6* shRNA was localized more diffusely to both cytoplasm and nuclei, and about the half of spheroids exhibited the mislocalization pattern of ezrin expression (Fig. 5a,d, Supplementary Fig. 7). Overexpression of FLAG-Uba6 in cells expressing anti-*UBA6* shRNA abrogated the phenotypes such as enlarged spheroids, the lack of lumen and diffuse subcellular localization of ezrin, indicating that the alterations in cellular morphology and ezrin expression resulted from Uba6 deficiency, not from off-target effects of the shRNA. Overexpression of FLAG-Uba6 in control cells minimally altered the morphology of epithelial acini and the expression pattern of ezrin (Fig. 5b–d). To assess whether deregulated expression of ezrin plays a role in perturbed lumen formation of Uba6-deficient MCF-10A spheroids, we generated MCF-10A cells expressing anti-*EZR* (ezrin) shRNA and cells co-expressing anti-*EZR* and anti-*UBA6* shRNAs. Anti-*EZR* shRNA alone did not significantly affect acinar morphogenesis in 3D culture, and 97% of cell spheroids exhibited lumen formation similar to the control (Fig. 6). About 70% of cell spheroids co-expressing anti-*EZR* and anti-*UBA6* shRNAs exhibited typical lumen formation in 3D culture, demonstrating substantial restoration of lumen formation in comparison with cells expressing anti-*UBA6* shRNA. These observations suggest that ectopic accumulation of ezrin is involved in the phenotype of Uba6 deficiency in epithelial morphogenesis. Taken together, Uba6-dependent control of ezrin polyubiquitination and stability is critical for proper subcellular localization of this polarity regulator and coordinated acinar morphogenesis of mammary epithelial cells.

## Discussion

Proteomic profiling of ubiquitinated proteins has been conducted in various cellular contexts and revealed the diverse roles for protein ubiquitination^32^,^33^,^34^,^35^,^36^,^37^. The present study demonstrates the first attempt to profile and differentiate the ubiquitination targets of the two E1 enzymes Uba1 and Uba6. The protein ubiquitination cascades in the cell consist of E1, E2 and E3 enzymes, with E3s playing a major role in determining the substrate specificity^38^. Since the two E1 enzymes interact with overlapping yet distinctive sets of E2s and each E2 may pair with its own set of E3s to mediate UB transfer^3^, we hypothesized that Uba1 and Uba6 may initiate distinctive E1-E2-E3 cascades to affect the specificity of protein ubiquitination. We evaluated the hypothesis by constructing the OUT cascades with xUba1 and xUba6 and screening for E1-specific substrates. The OUT screen is enabled by the strict orthogonality between the xUB-xE1 pairs and the native UB-E1 pairs (Fig. 2b). Such orthogonality allows xUB transfer through xUba1 or xUba6 to be superimposed on the background of native UB transfer, and cellular proteins with xUB conjugation can be determined without interference from native ubiquitination. Using this novel approach, we verified unique and overlapped sets of E2s for each E1 (Fig. 2d), and found that the two E1s have distinctive profiles of cellular ubiquitination targets (Fig. 2c). In contrast to the profiles of E2s, the OUT-based screens identified limited numbers of E3s (Supplementary Data 1–3). ARIH1, CBL, RNF40, RFFL and PAM/MYCBP2 were identified as Uba1-specific targets, while BIRC6, CHIP, RBBP6, TRIM25, TRIM51 and UHRF1 were identified as Uba6-specific targets. The previous study using HBT-wt UB expression in HeLa cells identified only 7 E3s (ref. 24, of which only UHRF1 overlaps with the E3s identified in the present study. The scarcity of E3s in the proteomic profiles may suggest their transient expression, low abundance and fast turnover in the cell. In addition, several deubiquitinating enzymes^39^ were identified as HBT-xUB-conjugated protein in our screens. UCHL1, UCHL3, UCHL5 and USP13 were identified only in the xUba1-mediated purification, and OTUB1, USP14, EIF3H, PRPF8 and PSMD14 were identified only in the xUba6-mediated purification. USP5 was found in the Uba6/Uba1-shared data set. These data provide new insight into the interplays of Uba1 and Uba6 with other components of the enzymatic systems for protein ubiquitination and deubiquitination.

The bioinformatics analysis of the potential Uba6-specific and Uba1-specific ubiquitination targets from the OUT screens suggested that Uba1 and Uba6 have non-redundant functions, which is consistent with the lethal phenotype of *Uba6* null mice^10^ and the cell cycle arrest induced by the temperature-sensitive allele of *Uba1* (refs 6, 7, 8, 9). It is noteworthy that IPA of the Uba6-specific targets lists synaptic long-term potentiation among uniquely associated pathways (Supplementary Fig. 4), which might be linked to the autism-like phenotype of mice with neuron-specific *Uba6* disruption^14^. Moreover, among the other pathways uniquely associated with Uba6-specific targets, six signalling pathways that involve Cdc42, RhoA, actin nucleation, integrin, epithelial adherens junction and tight junction are relevant to the developmental regulation of cell structure and motility, and suggest previously undefined roles of Uba6 in tissue development. Overall our findings suggest that the two E1 enzymes initiate ubiquitination of distinctive pools of substrates, through which they propagate unique signals across cellular regulatory networks.

Our study has verified that ezrin and CUGBP1 undergo Uba6-dependent polyubiquitination. The ezrin, radixin and moesin family consists of membrane/actin cytoskeleton linker proteins^26^,^27^. Ezrin forms plasma membrane-attached structures that control cell adhesion to the extracellular matrix, cell-cell interactions, cell morphology and motility. During epithelial morphogenesis, ezrin controls apical-basal polarity by organizing actin cytoskeletons and cell cortex structures^26^,^27^. Deregulation of ezrin is thought to play roles in cancer progression; abnormal ezrin distribution such as diffuse cytoplasmic and/or nuclear expression has been correlated with poor prognosis of breast cancer patients^40^,^41^,^42^. Furthermore, MDA-MB-231 breast cancer cells exhibit ezrin overexpression and silencing of ezrin can suppress their metastatic behaviours^43^. Our study demonstrated that Uba6-mediated ubiquitination of ezrin not only controls stability of the protein but also affects its subcellular localization. The analysis of MCF-10A cells in 3D culture demonstrated that Uba6 knockdown resulted in ectopic localization of ezrin in the cytoplasm and nucleus and also perturbed acinar formation, leaving many MCF-10A spheroids without typical lumen formation. In addition, Uba6-deficient cell spheroids were significantly enlarged. These phenotypes are reminiscent of the effects of oncoproteins such as ErbB2 and AKT (ref. 30). While the experiment with co-silencing ezrin and Uba6 (Fig. 6) suggested that perturbed control of ezrin ubiquitination played a role in impaired acinar morphogenesis of Uba6-deficient epithelial cells, ezrin knockdown rescued lumen formation of Uba6-deficient spheroids incompletely. These observations suggest that other Uba6-specific substrates are also involved in the phenotype, including those involved in regulation of epithelial cell polarity, proliferation and viability. Indeed, the Uba6-specific targets include a number of proteins that control development and oncogenesis, and one of such proteins is CUGBP1. This RNA binding protein controls multiple fates of RNAs such as alternative splicing, deadenylation, mRNA stability and translation^28^. A number of CUGBP1-associated mRNAs encodes regulators of cell growth, migration and apoptosis, such as p21^CDKN1A^, BAD and BAX (ref. 44), and high expression of CUGBP1 is an indicator of poor prognosis in cancer^45^,^46^. Future studies based on the substrate profile from the OUT screens are expected to identify more Uba6-specific targets that are important for epithelial morphogenesis and human diseases including neuronal disorders and cancer.

In this study, we engineered orthogonal xUB-xE1 pairs to distinguish the ubiquitination targets of Uba1 and Uba6 and their associated cell signalling pathways. The present study provides a compelling example on generating new biological insights by chemically manipulating protein recognition and enzyme catalysis in the cell. We expect the OUT cascade can be further extended to specific E2 and E3 enzymes in order to map the detailed structures of UB transfer networks beyond E1 and reveal the roles of individual E1-E2-E3 cascades in regulatory pathways that control diverse cellular functions.

## Methods

### Cell culture and reagents

Human non-transformed mammary epithelial MCF-10A cells and human embryonic kidney HEK293 cells were obtained from American Tissue Culture Collection (ATCC). Immortalized prostate epithelial RWPE-1 cells and prostate cancer PC-3M cells were described previously^47^ and obtained from Dr Sui Huang. All cells were cultured under standard conditions recommended by ATCC. Fetal bovine serum and horse serum were obtained from HyClone/Thermo Fisher Scientific (Logan, UT, USA), and media, antibiotics and other chemicals were purchased from Corning Cellgro (Manassas, VA, USA) and GiBCO/Invitrogen (Carlsbad, CA, USA). Cycloheximide was purchased from Sigma-Aldrich (St Louis, MO, USA).

### Primers

For plasmid construction:

Bo56: 5′-CGATACGACGCTAGCATGGAAGGATCCGAGCCTGTGGCC-3′

Bo57: 5′-AGTGATCAGCTCGAGTTAATCAGTGTCATGACTGAAGTAG-3′

Bo58: 5′-CGATACGACGCTAGCATGTCCAGCTCGCCGCTGTCCAAG-3′

Bo59: 5′-AGTGATCAGGCGGCCGCTCAGCGGATGGTGTATCGGACATAGGG-3′

Bo182: 5′-CAAATCTAAGGCCTCTTTTAGATTCTGGAACAATGGGCACTAAGGGACACACTCGAGTTATTGTACCGCATTTGACTGAGTCTTACAATAGTCGACGGCGACCCCCAGAAGAGGAAATA C-3′

Bo183: 5′- GGA AGGCTTGAATTCCTG-3′

Bo184: 5′-CGAGTGGTGATCCCCTTCCTGACAGAGTCGTACAGTTCCCGCCAGCGCCCACCTGAGAAGTCCATCCCC-3′

Bo185: 5′-CGACTCTGTCAGGAAGGGGATCACCACTCGCACATTGCCTTTGGTGCCCAG-3'

Bo186: 5′-GCTCAGCATGGCCGGCCACC-3′

Bo187: 5′-GCCAGACTTGGGGGTGAATTC-3′

### Construction of protein expression plasmids

The human Uba6 gene was amplified with primers Bo56 and Bo57, and the human Uba1 gene was amplified with primers Bo58 and Bo59. The amplified PCR products were then double digested by NheI and XhoI, and cloned into pET-28a plasmid for the expression of wt Uba1 and wt Uba6. To construct the xUba6 mutant, primers Bo182 and Bo183 were used to amplify fragments of the Uba6 gene with the incorporation of mutations E601R, H614R and D616R. The PCR fragment was digested with StuI and EcoRI restriction enzymes and cloned into the pET-wt Uba6 plasmid to generate pET-xUba6. To construct the xUba1 mutant, primers Bo184 and Bo185, and Bo 186 and Bo187 were paired to amplify the Uba1 gene in pET-wt Uba1. The amplified PCR fragment had mutations Q608R, S621R and D623R incorporated into the Uba1 gene. The two PCR fragments were assembled by overlapping PCR and cloned into the pET-wt Uba1 vector between restriction sites FseI and EcoRI to generate pET-xUba1.

The lentiviral vectors for Flag-UBA1, Flag-xUBA1, Flag-UBA6 and Flag-xUBA6 were generated in the pLenti-6 backbone with the blasticidin-resistance gene cassette. First, we made pLenti6-V5-D-TOPO-Asc1-Blasticdin vector, a 9bp-fragment (GGCGCGCCA) was inserted between nt2439 and nt2440 in the sequence of pLenti6-V5-D-TOPO plasmid (Invitrogen) by sub-cloning with BamH1/Xho1 enzyme sites. To make pLenti6-V5-D-TOPO-Asc1-Blasticidin-FLAG xUBA6/FLAG xUBA1 plasmids, the FLAG xUBA6/FLAG xUBA1 fragments were sub-cloned into pLenti6-V5-D-TOPO-Asc1-Blasticidin vector with Asc1 enzyme site. To make pLenti6-V5-D-TOPO-Asc1-hygromycin-HBT (x)UB plasmids, HBT tag was sub-cloned from pQCXIP HBT-Ubiquitin (26865, Addgene, Cambridge, MA, USA), which was developed by the Peter Kaiser laboratory^24^,^48^, and fused with DNA fragment of Human (x)Ub. The vector pLenti6-V5-D-TOPO-Asc1-hygromycin was obtained by replacing the blasticidin gene with hygromycin gene in pLenti6-V5-D-TOPO-Asc1-Blasticdin vector. The HBT-xUb fragments were subsequently sub-cloned into pLenti6-V5-D-TOPO-Asc1-hygromycin vector with BamH1/Asc1 enzyme sites.

### Expression and purification of the E1 enzymes from *E. coli*

For the expression of E1s, BL21 cells were transformed with the pET vector of E1 or xE1. A single colony of the transformed cells was inoculated into 5 ml of LB and the culture was grown overnight at 37 °C. The next day the culture was diluted into 1 l LB and grown at 30 °C until OD reaches ∼1. The culture was then induced for E1 expression with the addition of 4 mM IPTG. The culture was grown at 13 °C for 24 h before the cells were harvested. To purify E1 from the cell, cells were pelleted and resuspended in 50 ml lysis buffer containing 50 mM Tris pH 7.5, 500 mM NaCl, 5% glycerol and 5 mM β-mercaptoethanol (BME). One tablet of Roche complete mini protease inhibitor cocktail, 1 mM PMSF and 1 mM benzamidine were added to cell suspension. lysosome (1 mg ml^−1^) was also added to the cell suspension to lyse the cell wall polysaccharides. After being left on ice for 1 h, the cell suspension was sonicated to lyse the cells. The lysate was centrifuged and the supernatant was bound to Ni-NTA resin for 2 h. The resin was washed with 20 ml lysis buffer twice and eluted with 5 ml elution buffer (same as lysis buffer but with 250 mM imidazole). PMSF (1 mM) and benzamidine (1 mM) was also added to the lysis and elution buffer to prevent protein degradation.

### ATP-PP_i_ exchange assay

We used ATP/PP_i_ exchange assay to measure the kinetics of E1 activation of UB mutants^21^,^22^. In this assay, E1 catalyses the condensation of UB with ATP to form UB-AMP conjugate with the release of PP_i_. Externally added PP_i_ labelled with the radioactive isotope ^32^P sets the reaction in reverse thus incorporating ^32^PP_i_ into ATP. Radioactive ATP formed in the reaction is captured by charcoal and quantified. The rate of ^32^PP_i_ exchange into ATP reflects the reactivities of E1s with xUB or wt UB to form UB-AMP intermediates. To set up the reaction, 5 μM xUB or wt UB, and 0.5 μM E1 were added to a 50 μl reaction containing 50 mM Tris-Cl, pH 7.5, 10 mM MgCl_2_ and 1 mM ATP. The exchange reaction was initiated by adding 1 mM sodium [^32^P]pyrophosphate (4.6 Ci mol^−1^). The reaction was incubated at room temperature and quenched at various time points by addition of 0.5 ml of a suspension of activated charcoal (1.6% (w/v)) charcoal, 0.1 M tetrasodium pyrophosphate, and 0.35 M perchloric acid). After thorough mixing by vortex, the charcoal was pelleted by centrifugation and the pellet was washed by 1 ml 2% trichloroacetic acid for three times. Finally the charcoal pellet was resuspended in 0.5 ml water and 3.5 ml Ultima Gold LSC-cocktail (PerkinElmer) was added. The radioactivity bound to charcoal was determined by liquid scintillation counting.

### Lentivirus packaging and transduction

ViraPower Lentiviral Packaging Mix (K4975-00) was obtained from Life Technology. Virus packaging, virus infection and selection of stable cell lines were performed according to the manufacturer's protocol for the ViraPower Lentiviral Expression System. Lookout Mycoplasma PCR detection kit (Sigma, MP0035) was used to confirm the negative detection of mycoplasma contamination of all cell lines used for the study.

### Lentiviral silencing of *UBA6* and *UBA1*

Lentiviral GPIZ plasmids encoding shRNAs against UBA6 and UBA1 (five different shRNAs against each gene) were obtained from GE Dharmacon (Lafayette, CO, USA), and lentiviruses were produced using the manufacturer's lentivurus packaging system and 293FT cells. HEK293 cells were infected with each lentivirus, followed by selection with puromycin for stable cell populations. Efficiency of gene silencing in each shRNA group was determined by immunoblotting using stable cell populations. For functional restoration, HEK293 cell population stably expressing anti-UBA6 shRNA (clone ID: V2LHS_262958) were further infected with the lentivirus packaged with pLenti6-Flag-tagged wild-type UBA6, and selected with puromycin and blasticidin.

### Immunoblotting

To assay xUB transfer to xE1, 10 μM HA tagged xUB or wt UB was added into 100 μl reaction with 1 μM E1 (wt Uba6, wt Uba1, xUba6 or xUbe1), 10 μM E2 (UbcH5b) and 6 μM E3 (CHIP), 1 mM ATP, 50 mM MgCl_2_ and 50 μM dithiothreitol (DTT) in TBS buffer (20 mM Tris HCl, 150 mM NaCl, pH7.5) for 1 h at room temperature before SDS-PAGE and western blot analysis. Twenty microlitres reaction was loaded on the SDS-PAGE gel (Bio-Rad, Hercules, CA, USA) for protein separation by electrophoresis. The protein bands on the gel were blotted on a PVDF membrane (Bio-Rad). The membrane was blocked with 5% milk in TBS-T buffer (0.05 % (v/v) Tween 20, 0.05 % (v/v) Triton X-100 in TBS, pH7.5) for 1 h, then incubated with 5% milk TBS-T buffer (pH7.5) containing 1:500 diluted 200 μg ml^−1^ anti-HA antibody (Santa Cruz Biotechnology, Dallas, TX, USA) and 1:10,000 diluted anti-mouse horseradish peroxidase conjugate (Thermo Scientific, Rockford, IL, USA) for 1 h, respectively. The membrane was washed five times by TBS-T buffer (pH7.5) and five times by TBS buffer (pH7.5) and then detected with ECL luminescent detection kit (GE Healthcare, Little Chalfont, Buckinghamshire, UK). Immunoblotting using mammalian cell lysates was performed using NP40-based lysis buffer (50 mM HEPES/KOH pH7.5, 150 mM NaCl, 1 mM EDTA, 2.5 mM EGTA, 1 mM DTT, 10 mM β-glycerophosphate, 1 mM NaF, 0.1 mM Na_3_VO_5_, 0.1 % (v/v) NP-40, 10 % (v/v) Glycerol and cOmplete Protease Inhibitor Cocktail, Sigma-Aldrich, St Louis, MO, USA)^49^. Unless otherwise specifically indicated, the primary antibodies and secondary antibodies were 1:1,000 and 1:3,000 diluted, respectively. Anti-His antibody was obtained from Qiagen (34660, Valencia, CA, USA), Anti-FLAG antibody (sc-807, 1:600 dilution), anti-Ezrin antibody (sc-58758), anti-Ub antibody (sc-8017) and anti-CUGBP1 mouse monoclonal antibody (sc-20003) were obtained from Santa Cruz Biotechnology (Dallas, TX, USA). Anti-UBA6 mouse monoclonal antibody (H00055236-M08) and anti-CUGBP1 rabbit polyclonal antibody were obtained from Abnova (H00010658-D01P, Walnut, CA, USA). Anti-UBA6 rabbit polyclonal antibody (AP16886b-ev) and anti-UBA1 antibody were obtained from Abgent (AP14555a-ev, San Diego, CA, USA). Anti-α-tubulin antibody was purchased from Sigma-Aldrich (T6199, 1:8,000 or 1:20,000 dilution). horse radish peroxidase conjugated streptavidin was purchased from Life Technology (21126, Carlsbad, CA, USA, 1:2,000 dilution). Target proteins were visualized by enhanced chemiluminescence (Thermo Scientific, Rockford, IL, USA). Uncropped original blots are shown in Supplementary Figs 8–14. The band intensities were quantified by densitometry using ImageQuant and normalized to those of their respective control bands. Data were expressed as fold changes compared with an appropriate control.

### Co-immunoprecipitation

For immunoprecipitation, cells were lysed by sonication in lysis buffer as described previously^49^. Unless otherwise noted, 50 μg total protein lysate was loaded onto gel. For immunoprecipitation with anti-CUGBP1 or ezrin antibody, 1 mg protein lysates were incubated with 1 μg relevant antibody overnight at 4 °C, followed by incubation with protein A or protein G for 1 h at 4 °C. The expression vector for wild-type UB pCMV-6His-HA-Ubiquitin was obtained from Dr Antonio Iavarone, Columbia University, as a kind gift. The expression vector for UB mutant pRK5-HA-Ubiquitin-KO (17603), pRK5-HA-Ubiquitin-K11 (22901), pRK5-HA-Ubiquitin-K48 (17605), pRK5-HA-Ubiquitin-K29R (17602) and pRK5-HA-Ubiquitin-K63 (17606) plasmids were purchased from Addgene. Plasmid transfection was conducted with the Lipofectamine 2000 reagent from Invitrogen (11668-019, Carlsbad, CA, USA), according to the manufacturer's protocol. For CUGBP1 or ezrin immunoprecipitation for ubiquitination detection, cells were treated with 10 μM MG132 (American Peptide, Sunnyvale, CA, USA) for 90 min at 72 h post-transfection. Half of the immunoprecipitates were loaded onto one gel, gel was transferred onto nitrocellulose membrane as usual, then prepared for Ubiquitin blotting by boiling the membrane for 10 min.

### Tandem affinity purification of ubiquitinated proteins

Tandem purification was performed in at least three biological replicates for each condition. HEK293 cells (30 × 100 mm dishes) stably expressing FLAG-xE1s were acutely infected with lentivirus HBT-xUB for 72–86 h. To inhibit proteasome activity, cells were treated with 10 μM MG132 for 90 min at 37 °C. Cells were washed twice with ice cold 1 × PBS, pH 7.4, and harvested by cell scraper with buffer A (8 M urea, 300 mM NaCl, 50 mM Tris, 50 mM NaH_2_PO_4_, 0.5% NP-40, 1 mM PMSF and 125 U ml^−1^ Benzonase, pH 8.0)^48^. For Ni-NTA purification: cell lysates were centrifuged at 15,000*g* for 30 min at room temperature. Thirty-five microlitres of Ni^2+^ Sepharose beads (GE Healthcare) for each 1 mg of protein lysates were added to the clarified supernatant. After incubation overnight at room temperature in buffer A with 10 mM imidazole on a rocking platform, Ni^2+^ Sepharose beads were pelleted by centrifugation at 100*g* for 1 min and washed sequentially with 20-bead volumes of buffer A (pH 8.0), buffer A (pH 6.3) and buffer A (pH 6.3) with 10 mM imidazole. After washing the beads, proteins were eluted twice with five bead volumes of buffer B (8 M Urea, 200 mM NaCl, 50 mM Na_2_HPO_4_, 2% SDS, 10 mM EDTA, 100 mM Tris, 250 mM imidazole, pH 4.3). For streptavidin purification: the pH of above elution was adjusted to pH 8.0. Two microlitres of streptavidin agarose beads (Thermo Scientific, Rockford, IL, USA) for each 1 mg of initial protein lysate were added to the elution to bind ubiquitinated proteins. After incubation on a rocking platform overnight at room temperature, streptavidin beads were pelleted and washed sequentially with 2 × 25 bead volumes of buffer C (8 M Urea, 200 mM NaCl, 2% SDS, 100 mM Tris, pH 8.0), buffer D (8 M Urea, 1.2 M NaCl, 0.2% SDS, 100 mM Tris, 10% EtOH, 10% Isopropanol, pH 8.0) and buffer E (8 M urea, 100 mM NH_4_HCO_3_, pH 8). Reduction, alkylation and Two-Step In-Solution Digestion were performed according to the protocol of Trypsin/Lys-C Mix (Promega, Fitchburg, WI, USA).

### LC-MS/MS–LTQ Orbitrap velos-northwestern

LC-MS/MS was performed generally as previously described^24^. After on bead digestion, the beads were washed four times with buffer A (5% acetonitrile 0.1% formic acid buffer). All the washes were pooled and were desalted using reverse phase C18 spin columns (Thermo Fisher Scientific, Rockford, IL, USA). After desalting the peptides were concentrated to dryness in vaccuo. After drying the peptides were suspended in 5% acetonitrile and 0.1% formic acid. The samples were loaded directly onto a 15 cm long, 75 μM reversed phase capillary column (ProteoPep II C18, 300 Å, 5 μm size, New Objective, Woburn, MA, USA) and separated with a 200 min gradient from 5% acetonitrile to 100% acetonitrile on a Proxeon Easy n-LC II (Thermo Scientific, San Jose, CA, USA). The peptides were directly eluted into an LTQ Orbitrap Velos mass spectrometer at Northwestern University (Thermo Scientific, San Jose, CA, USA) electrospray ionization at 350 nl min^−1^ flowrate. The LTQ Orbitrap Velos mass spectrometer was operated in data dependent mode, and for each MS1 precursor ion scan the ten most intense ions were selected from fragmentation by CID (collision induced dissociation). The other parameters for mass spectrometry analysis were: resolution of MS1 was set at 60,000, normalized collision energy 35%, activation time 10 ms, isolation width 1.5, and +4 and higher charge states were rejected. The spectra were searched using Proteome Discover 1.4 with parameters as follows: (i) enzyme specificity: trypsin; (ii) fixed modification: cysteine carbamidomethylation; (iii) variable modification: methionine oxidation and N-terminal acetylation; (iv) precursor mass tolerance was ±10 p.p.m.; and (v) fragment ion mass tolerance was ±0.8 Da. All the spectra were searched against target/decoy databases and results were used to estimate the *q* values in Percolator algorithm as embedded in Proteome discoverer 1.4. The peptide identification was considered valid at *q* value<0.1 and peptides were grouped for protein inference to satisfy the rule of parsimony. Further, in the final protein list protein identification was considered only valid if supported by minimum of one unique peptide and final protein level false discovery rate was 1%.

### LC-MS/MS–Orbitrap fusion–emory

Bead solutions were treated with 1 mM DTT at 25 °C for 30 min, followed by 5 mM iodoacetimide at 25 °C for 30 min in the dark. Samples were digested with 1:100 (w/w) lysyl endopeptidase (Wako) at 25 °C for 2 h and further digested overnight with 1:50 (w/w) trypsin (Promega) at 25 °C, as described previously^50^. Resulting peptides were desalted with a Sep-Pak C18 column (Waters) and dried under vacuum. Derived peptides were resuspended in peptide 20 μl of loading buffer (0.1% formic acid, 0.03% trifluoroacetic acid, 1% acetonitrile). Peptide mixtures (6 μl) were separated on a self-packed C18 (1.9 μm Dr Maisch, Germany) fused silica column (25 cm × 75 μM internal diameter (ID); New Objective, Woburn, MA, USA) by a Dionex Ultimate 3000 RSLCNano and monitored on a Orbitrap Fusion mass spectrometer (Thermo Fisher Scientific , San Jose, CA, USA). Peptides were loaded onto the column in 1% buffer B at a flowrate of 300 nl min^−1^ for 25 min. Elution was then performed over a 140 min multistep gradient at a rate of 300 nl min^−1^ with buffer B ranging from 3 to 99% (buffer A: 0.1% formic acid in water, buffer B: 0.1 % formic in acetonitrile). The mass spectrometer was programmed to collect at the top speed for three second cycles. The MS scans (400–1,600 m/z range, 200,000 AGC, 50 ms maximum ion time) were collected at a resolution of 120,000 at m/z 200 in profile mode and the HCD MS/MS spectra (2 m/z isolation width, 30% collision energy, 10,000 AGC target, 35 ms maximum ion time) were detected in the ion trap. Dynamic exclusion was set to exclude previous sequenced precursor ions for 20 s within a 10 p.p.m. window. Precursor ions with +1, and +8 or higher charge states were excluded from sequencing. Spectra were searched using the Proteome Discoverer 2.0 platform with an embedded SEQUEST HT search engine (Thermo Scientific, San Jose, CA, USA) against human Uniprot database (90,300 target sequences). Searching parameters included fully tryptic restriction and a parent ion mass tolerance (± 20 p.p.m.). Methionine oxidation (+15.99492 Da), asparagine and glutamine deamidation (+0.98402 Da), lysine ubiquitination (+114.04293 Da) and protein N-terminal acetylation (+42.03670) were variable modifications (up to three allowed per peptide); cysteine was assigned a fixed carbamidomethyl modification (+57.021465 Da). Percolator was used to filter the peptide spectrum matches to a false discovery rate of 1%.

### Data prioritization using the CRAPome database

The CRAPome database consists of data from negative control experiments for affinity purification-mass spectrometry studies^25^. Proteins identified by LC-MS/MS were analysed by the workflow 1 of the database for sorting in the order of frequencies of detection in negative control experiments, and those in the category of most frequently detected proteins^25^ were grouped as lower priority (highlighted in grey shade, Supplementary Data 1–3). It should be noted that CRAPome is based on data from interactome studies, whose affinity purification was conducted under non-denaturing conditions. In contrast, the tandem purification for OUT screens was performed under more stringent denaturing conditions. Nonetheless, most frequently detected proteins in the CRAPome database are highly abundant proteins that can attach to resins largely in bait-independent manners^25^ and some of them might be detected nonspecifically even under denaturing conditions in the OUT screens.

### Analysis of ubiquitination sites

The localization of each ubiquitination site was evaluated by detection of diGly-containing peptides, as described previously^34^,^37^. Data across all experiments were used to generate a non-redundant list of observed diGly sites. In some experiments diGly-containing peptides were enriched using the PTMScan Ubiquitin Branch Motif (K-ɛ-GG) Immunoaffinity Beads (Cell Signaling Technology, Danvers, MA, USA), following tandem affinity purification and trypsin digestion. The mUbiSiDa database^51^ was used to list known ubiquitination sites for each protein identified in the present study. In Supplementary Data 1–3, ubiquitination sites detected in the present study are highlighted in blue fonts, and those sites that had not been archived in mUbiSiDa are indicated by italicized blue fonts.

### Pathways and networks analysis

IPA software (http:/www.ingenuity.com) was used to map and identify the biological networks and molecular pathways with a significant proportion of genes having Uba1-specific, Uba6-specific and Uba1/Uba6-shared ubiquitination targets respectively. Fisher exact test in IPA software was used to calculate *P* values for pathways and networks. The level of statistical significance was set at a *P* value <0.05. Pathways that are composed of 26 or fewer proteins were excluded from the heat map analysis in Supplementary Fig. 4.

### Immunofluorescence microscopy

MCF-10A cells were cultured in 3D using Matrigel, as previously demonstrated^52^. Cell cultures in chamber slides were fixed with 4% paraformaldehyde, followed by staining for immunofluorescence using anti-ezrin antibody (3C12) from Santa Cruz Biotechnology, Santa Cruz, CA, USA and Alexa 488-conjugated anti-mouse IgG, Alexa 568-conjugated phalloidin, and Hoechst 33342 from Life Technologies, Grand Island, NY, USA. Samples were then analysed by Nikon C2 Confocal Microscope. For quantification of spheroid sizes, images in × 10 magnification were acquired by TissueFAXS 200 (Tissuegnostics, Vienna, Austria), followed by analysis with the Image J software.

### Data availability

Data supporting the findings of this study are available within the article, and its Supplementary Information files, and from the corresponding author on reasonable request. The MS raw data have been deposited to the ProteomeXchange Consortium (http://proteomecentral.proteomexchange.org) via the PRIDE partner repository with the data set identifiers PXD005500 and PXD005513.

## Additional information

**How to cite this article:** Liu, X. *et al*. Orthogonal ubiquitin transfer identifies ubiquitination substrates under differential control by the two ubiquitin activating enzymes. *Nat. Commun.* **8,** 14286 doi: 10.1038/ncomms14286 (2017).

**Publisher's note:** Springer Nature remains neutral with regard to jurisdictional claims in published maps and institutional affiliations.

## Supplementary Material

Supplementary Information Supplementary Figures

Supplementary Data 1 Uba6-specific ubiquitination substrates identified by the Orthogonal Ubiquitin Transfer-based screens

Supplementary Data 2 Uba1-specific ubiquitination substrates identified by the Orthogonal Ubiquitin Transfer-based screens

Supplementary Data 3 Ubiquitination substrates shared by Uba6- and Uba1-dependent enzymatic cascades. These proteins appeared in both xUba6- and xUba1-based Orthogonal Ubiquitin Transfer screens.

Supplementary Data 4 Identities of proteins purified from HEK293 cells expressing only HBT-xUB

Supplementary Data 5 Ingenuity canonical pathways listed in Supplementary Figure 4

Supplementary Data 6 Protein networks associated with Uba6-specific, Uba1-specific, and Uba6/Uba1-shared ubiquitination targets
