# Supplementary material for: Orthogonal ubiquitin transfer identifies ubiquitination substrates under differential control by the two ubiquitin activating enzymes
Source: Nat Commun. 2017 Jan 30;8:14286. doi: 10.1038/ncomms14286 (PMC5290280; doi:10.1038/ncomms14286)
Supplement: Supplementary Information — Supplementary Figures [file ncomms14286-s1.pdf]

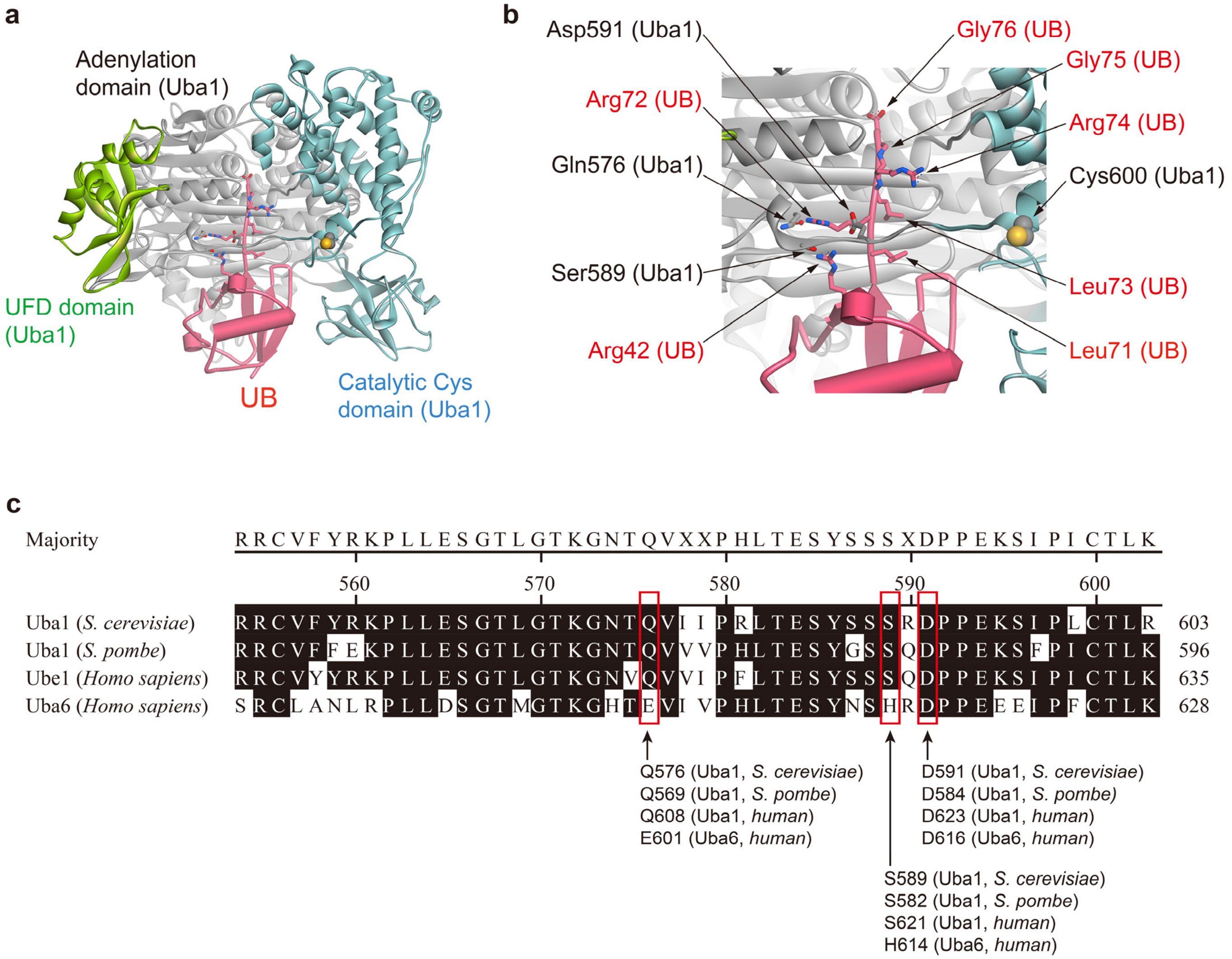

Supplementary Figure 1. (a) Three dimensional structure of the UB-Uba1 complex with the adenylation domain of yeast Uba1 in grey, and ubiquitin in red (PDB ID 3CMM[19]). **(b)** Key interactions between the UB C-terminal residues and the adenylation domain of Uba1. Cys600 forms thioester bond with the C-terminal carboxylate group of UB. **(c)** Alignment of peptide sequences in the adenylation domain of Uba1 and Uba6. Residues mutated in xUba1 and xUba6 were shown in red frames.

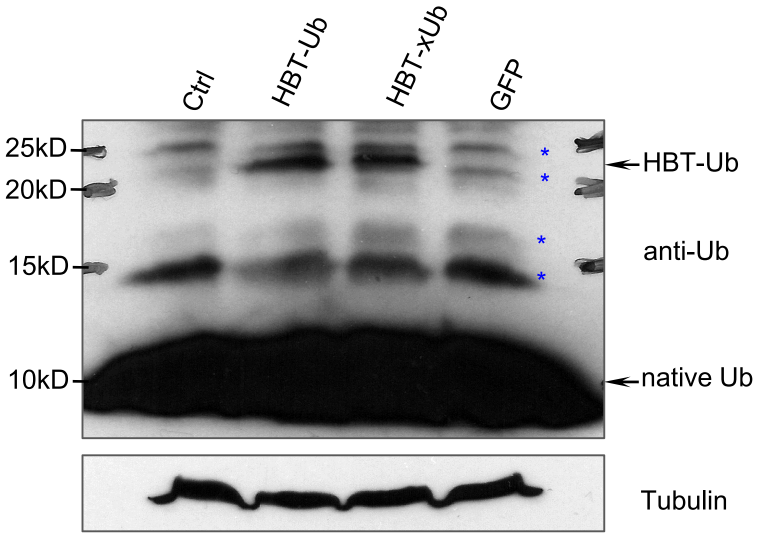

Supplementary Figure 2. Comparison of the expression of HBT-(x)UB and native UB. The same blot in the bottom left panel in Fig. 2b was re-probed with anti-UB antibody. Ctrl, parental HEK293 cells without viral transduction; GFP, cells infected with a lentivirus for green fluorescent protein.

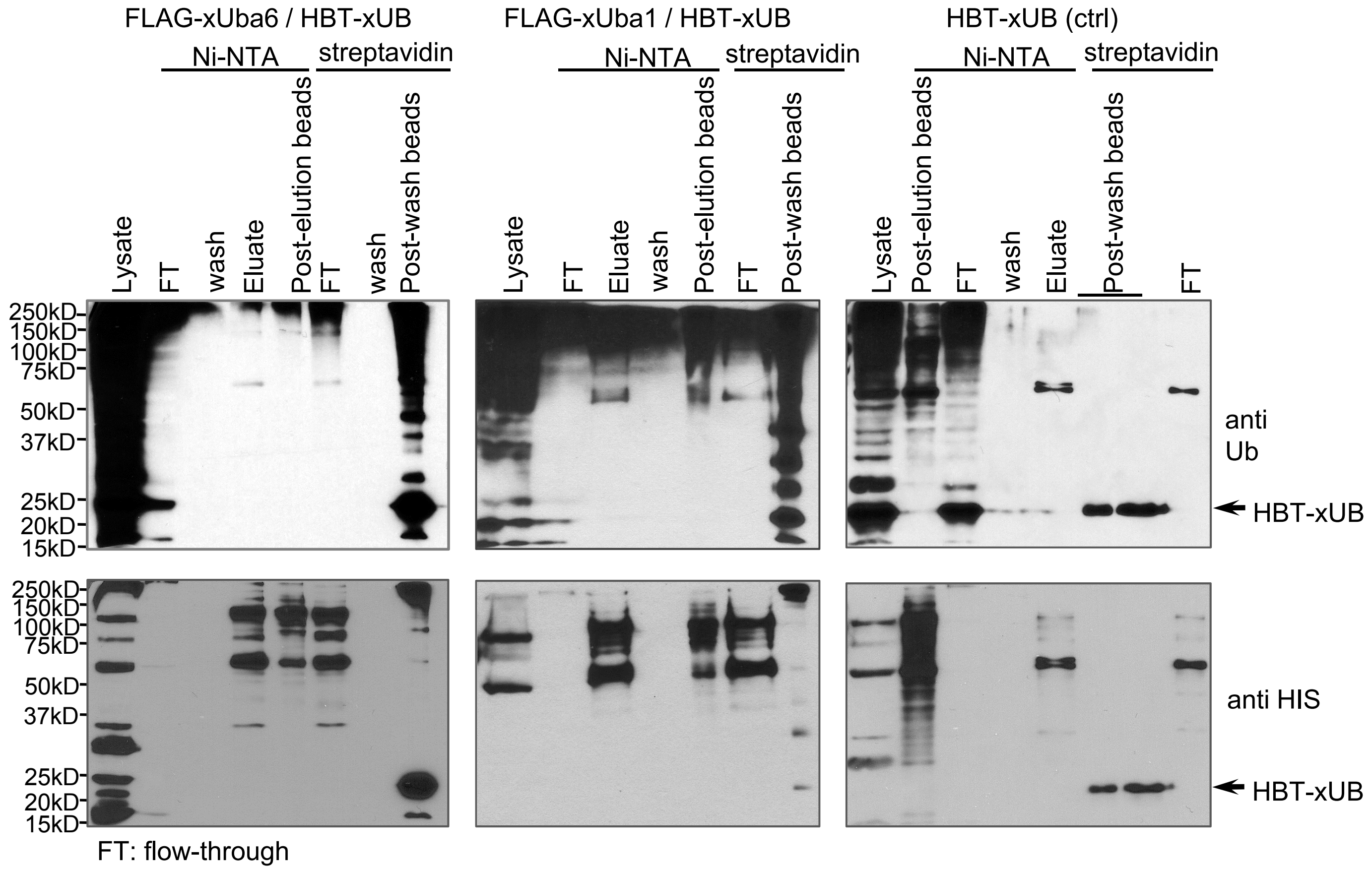

Supplementary Figure 3. Tandem purification of HBT-xUB-conjugated proteins from HEK293 cells stably expressing HBT-xUB and FLAG-xUba6 or FLAG-xUba1. Samples at the indicated steps of tandem affinity purifications were analyzed by immunoblotting using anti-His or anti-ubiquitin (UB) antibody.

Supplementary Figure 4

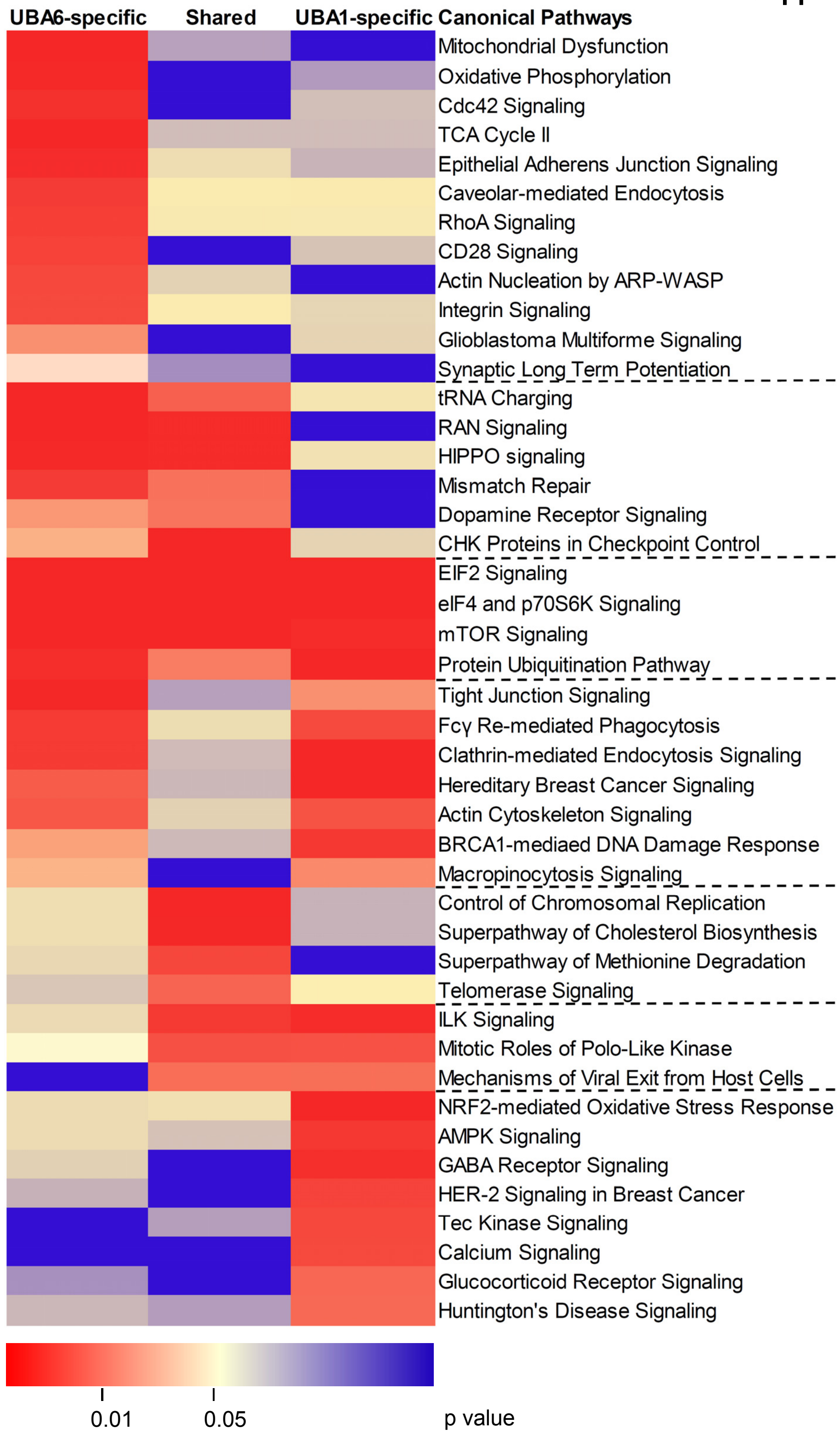

Supplementary Figure 4. Ingenuity Canonical Pathways (IPA) analyses of the pathways that are significantly associated with the Uba6-specific, Uba1-specific and/or Uba6/Uba1-shared ubiquitination substrates. The heat map demonstrates statistical significance of association of each pathway with the Uba6-specific, Uba6/Uba1-shared, and Uba1-specific substrates (see the bottom chart for the color scheme displaying p values). The pathways are categorized into seven groups (separated by broken lines), according to statistically significant ($p<0.05$) association with the three datasets. For complete lists of protein components of the canonical pathways, see Supplementary Data 5.

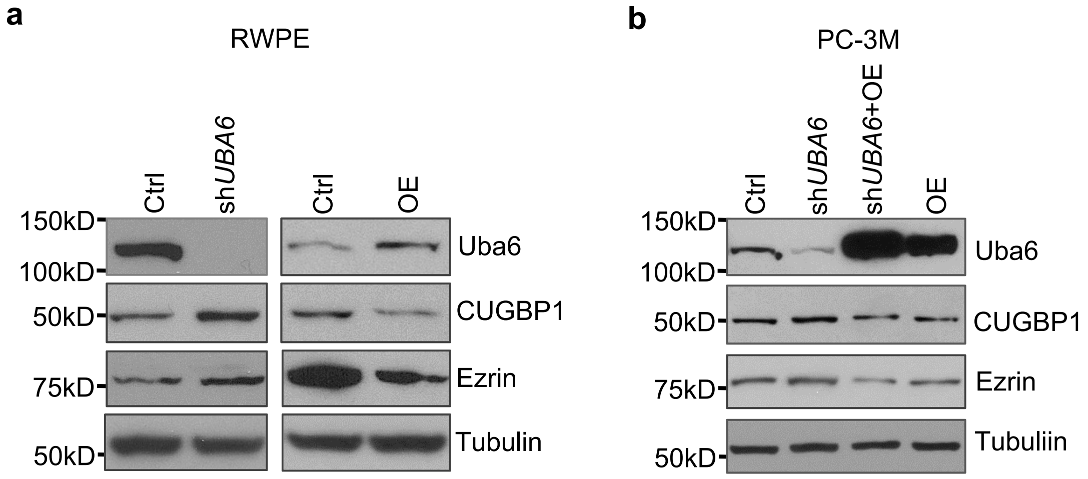

Supplementary Figure 5. Effects of anti-*UBA6* shRNA or Uba6 overexpression (OE) on cellular levels of the two Uba6-specific ubiquitination targets, ezrin and CUGBP1 in RWPE-1 human prostate epithelial cells (**a**) and in PC-3M human prostate cancer cells (**b**). The indicated cell lines were infected with recombinant lentivirus for the shRNA or cDNA, drug-selected, and harvested for immunoblotting.

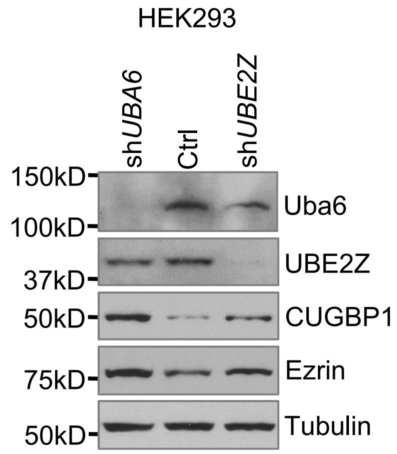

Supplementary Figure 6. Effects of anti-*UBE2Z* shRNA on cellular levels of the two Uba6-specific ubiquitination targets, ezrin and CUGBP1, in HEK293 cells, in comparison with those of anti-*UBA6* shRNA. The indicated cell lines were infected with recombinant lentivirus for the shRNA or cDNA, drug-selected, and harvested for immunoblotting.

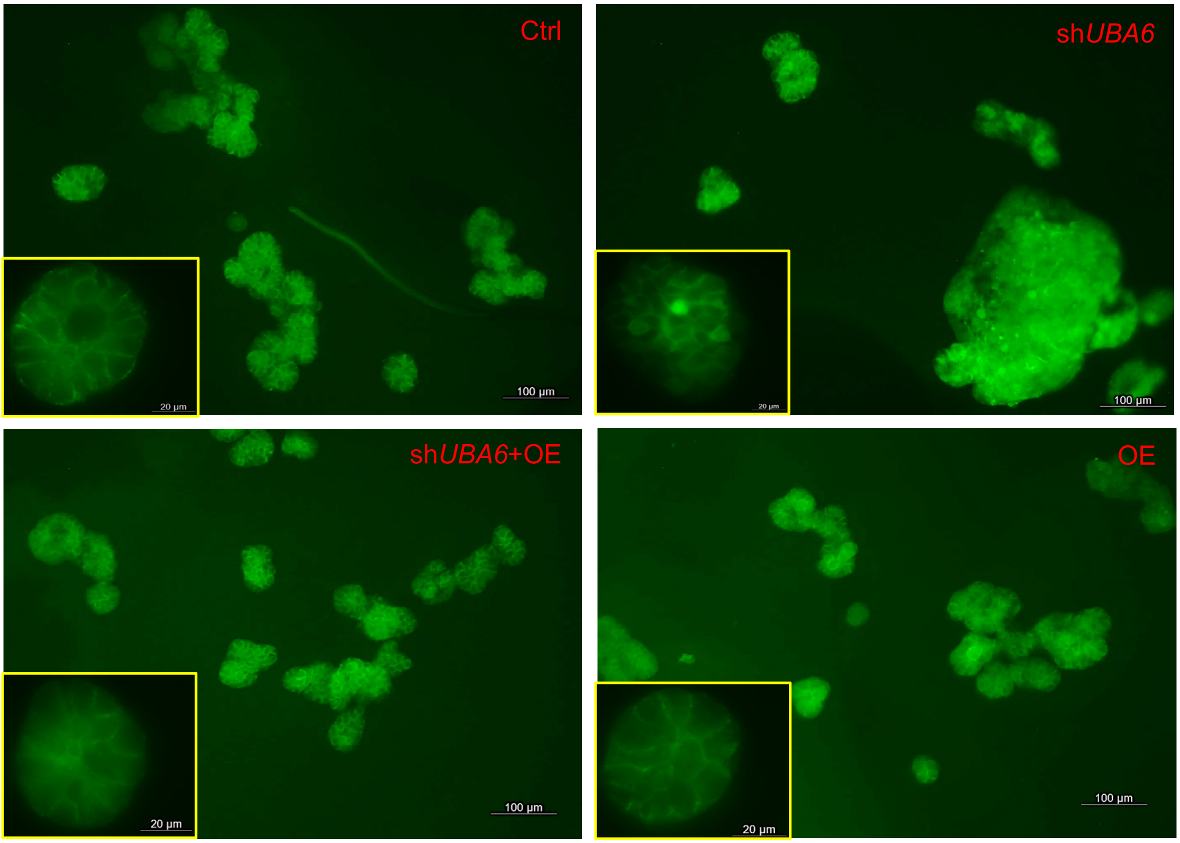

Supplementary Figure 7. Effects of *UBA6* silencing or overexpression on the sizes and shapes of MCF-10A cell spheroids in 3-D cultures and on subcellular expression patterns of ezrin. Representative images (10X and 40X objective lens, FITC channel, 14d 3D culture stained with Ezrin) are shown to display the formation of large cell aggregates without typical lumen structures and cytoplasmic mislocalization of ezrin in cells expressing anti-*UBA6* shRNA (*shUBA6*) (upper right panels). Forced overexpression (OE) of Uba6 in cells expressing *shUBA6* restored the formation of acinar-like spheroids with typical lumens and enrichment of ezrin expression on the plasma membrane (lower left panels). Immunofluorescence microscopy was conducted after staining with anti-ezrin monoclonal antibody and Alexa-488-conjugated secondary antibody.

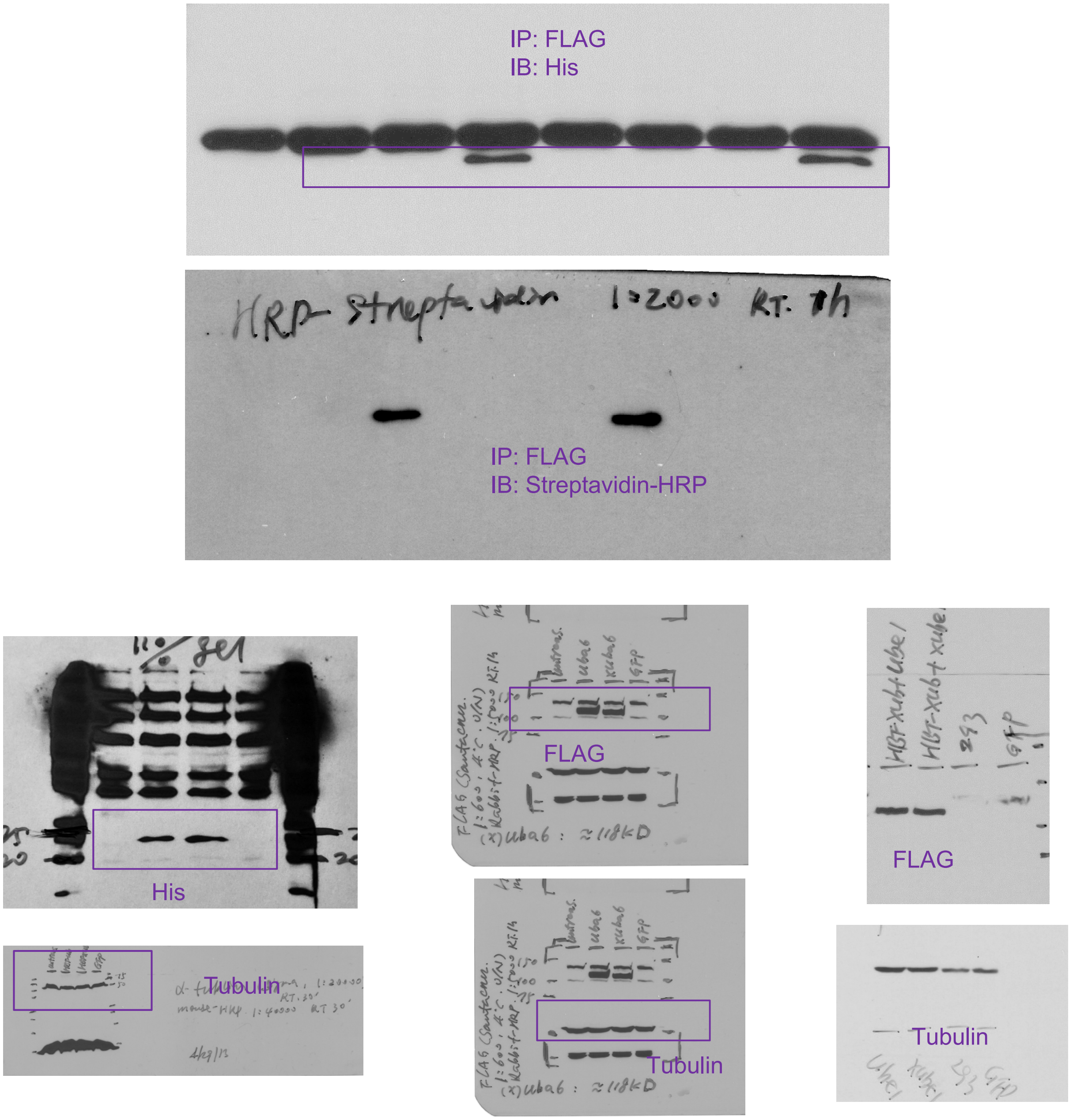

Supplementary Figure 8. Uncropped immunoblots for Fig. 2b.The purple squares indicate the sections shown in Fig. 2b.

Supplementary Figure 9a

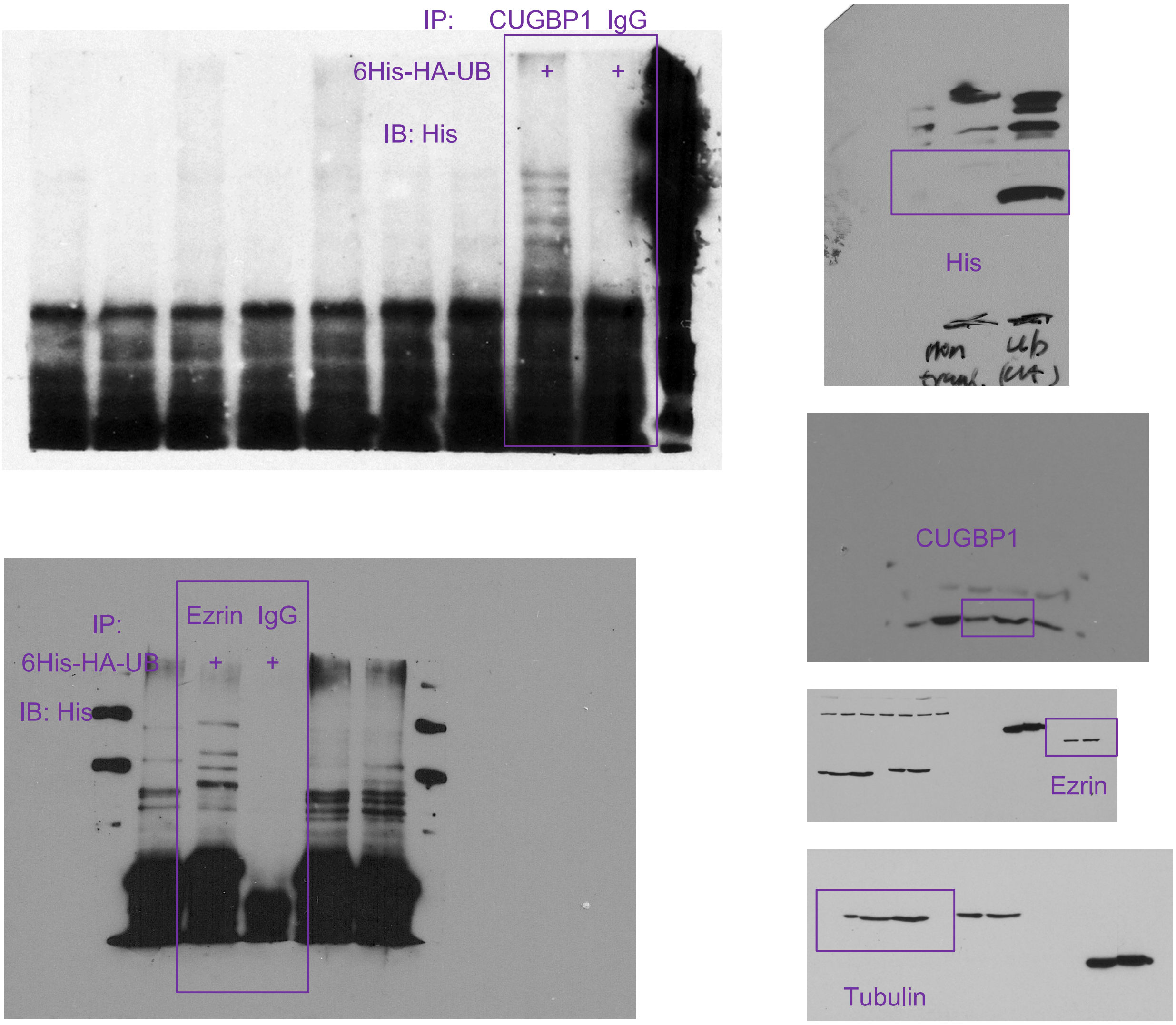

Supplementary Figure 9a. Uncropped immunoblots for Fig. 3a. The purple squares indicate the sections shown in Fig. 3a.

Supplementary Figure 9b

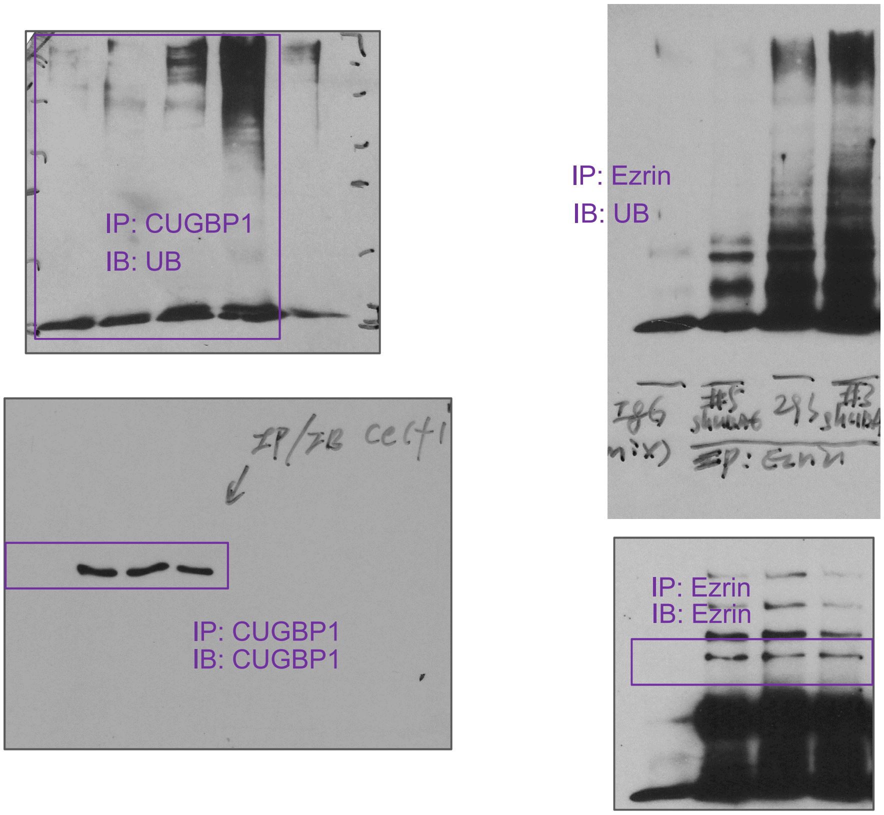

Supplementary Figure 9b. Uncropped immunoblots for Fig. 3b. The purple squares indicate the sections shown in Fig. 3b.

Supplementary Figure 10a

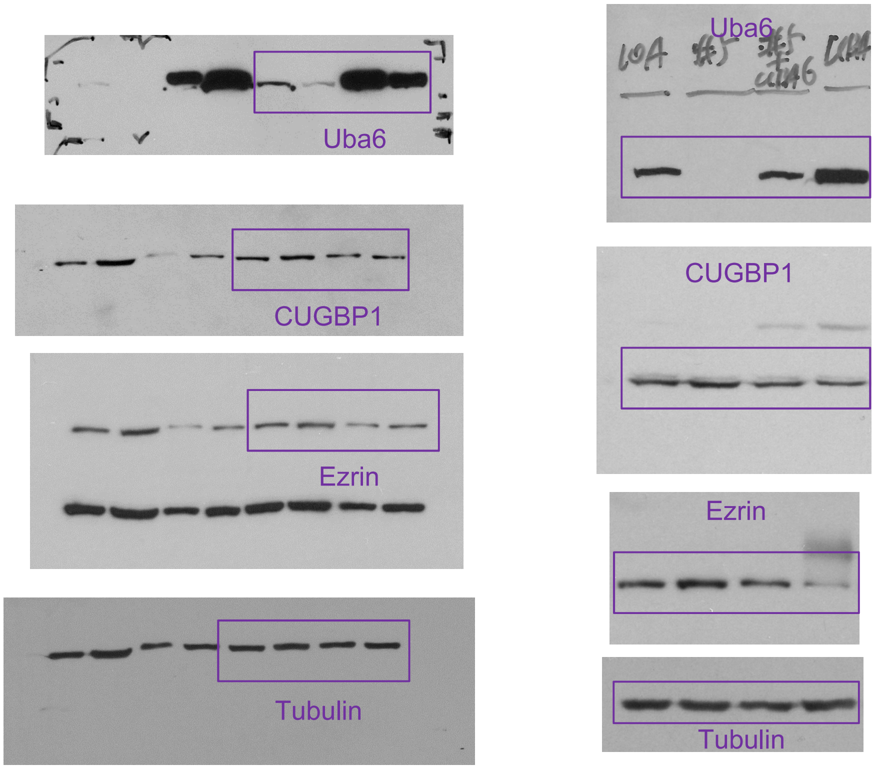

Supplementary Figure 10a. Uncropped immunoblots for Fig. 4a. The purple squares indicate the sections shown in Fig. 4a.

Supplementary Figure 10b

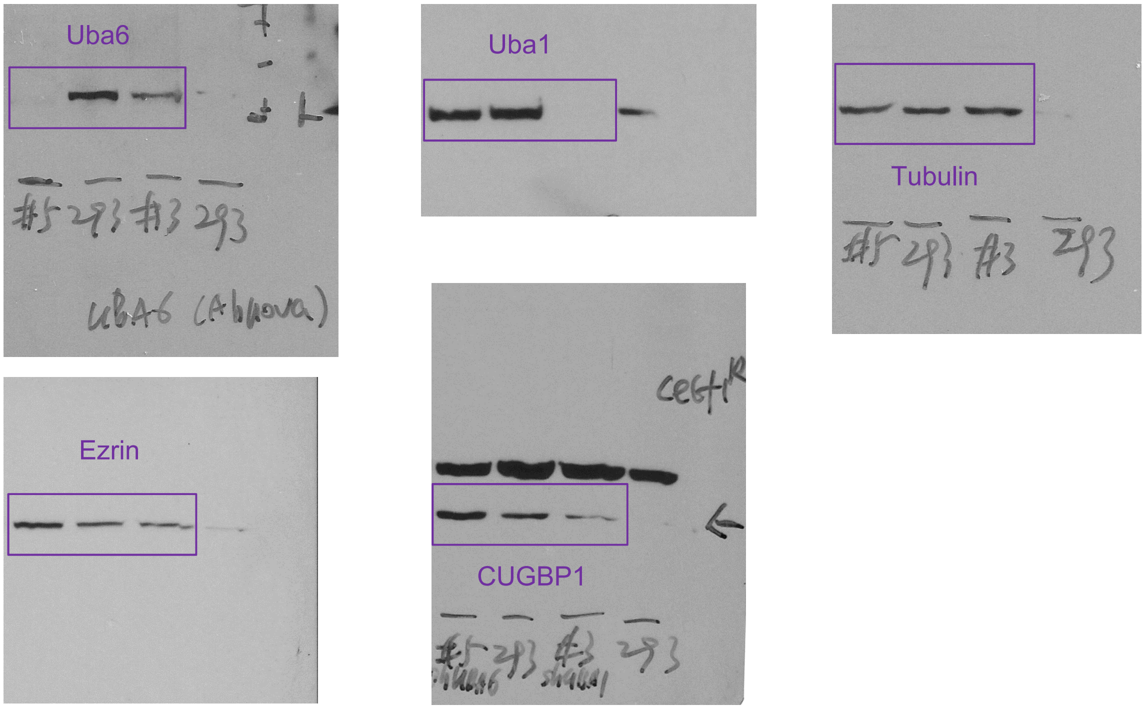

Supplementary Figure 10b. Uncropped immunoblots for Fig. 4b. The purple squares indicate the sections shown in Fig. 4b.

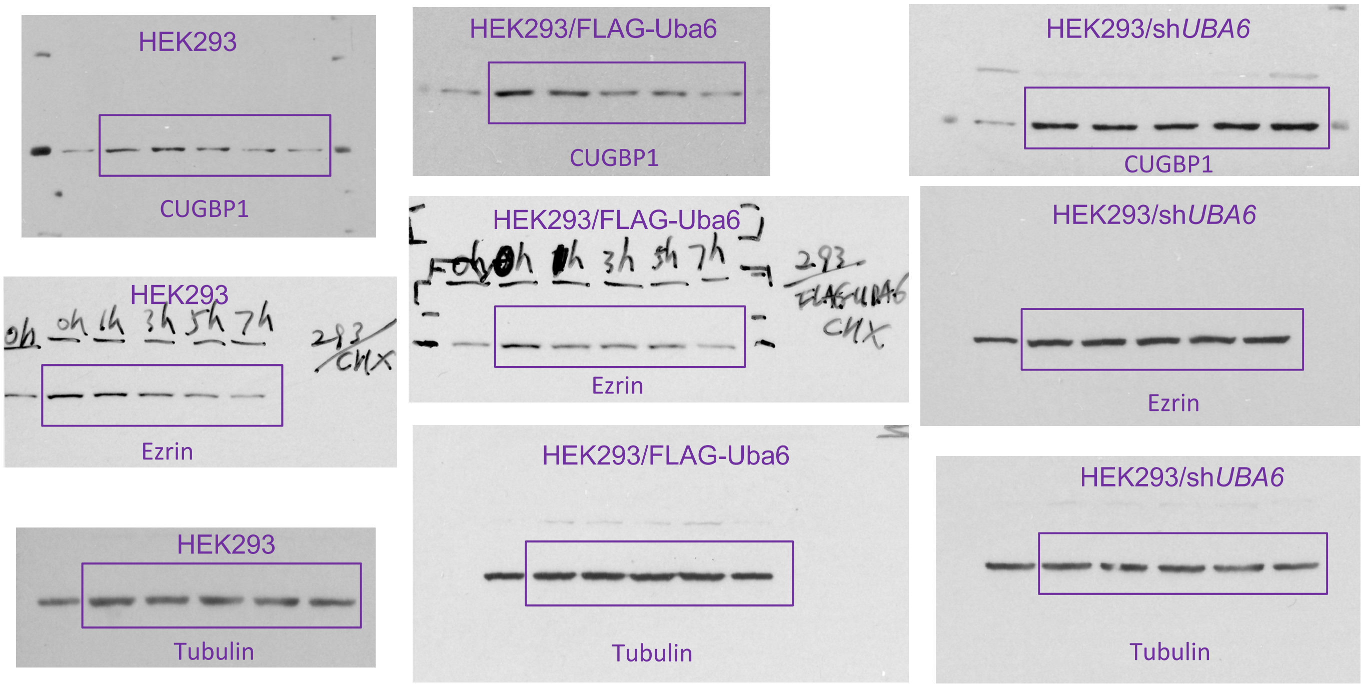

Supplementary Figure 10c. Uncropped immunoblots for Fig. 4c. The purple squares indicate the sections shown in Fig. 4c.

Supplementary Figure 10d

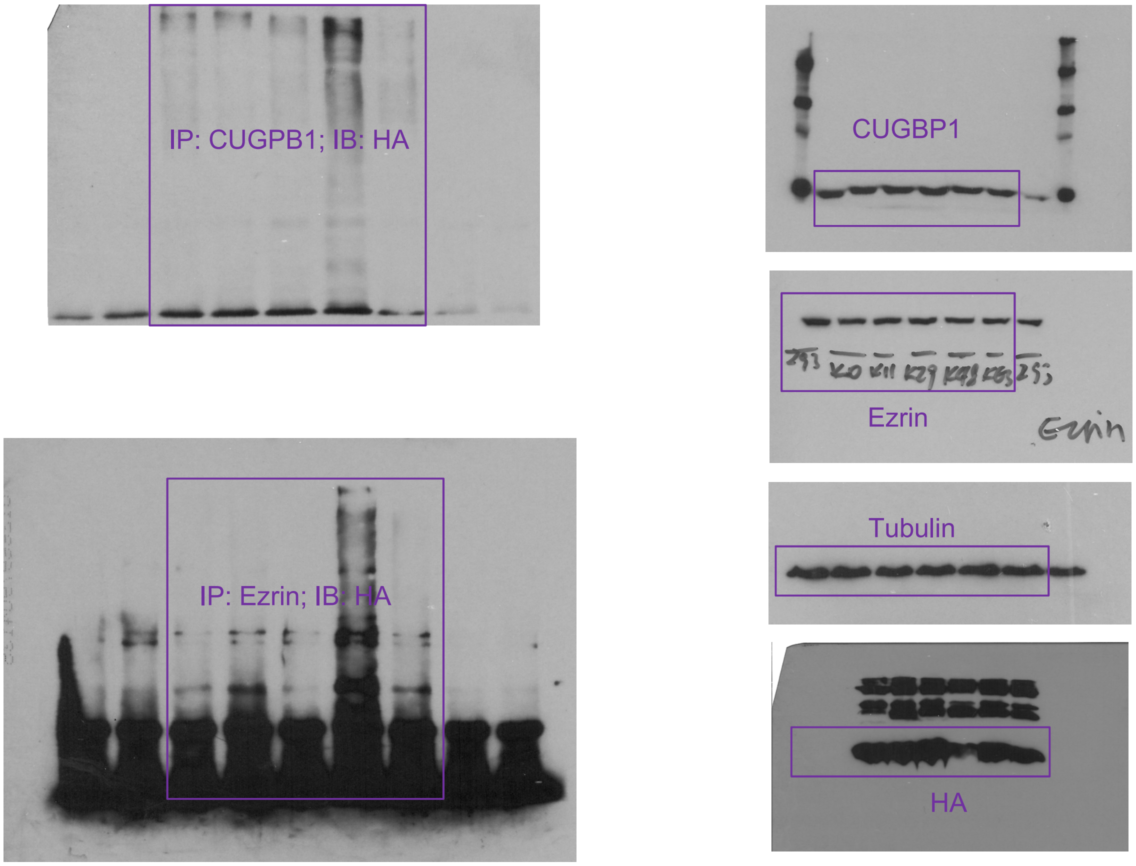

Supplementary Figure 10d. Uncropped immunoblots for Fig. 4d. The purple squares indicate the sections shown in Fig. 4d.

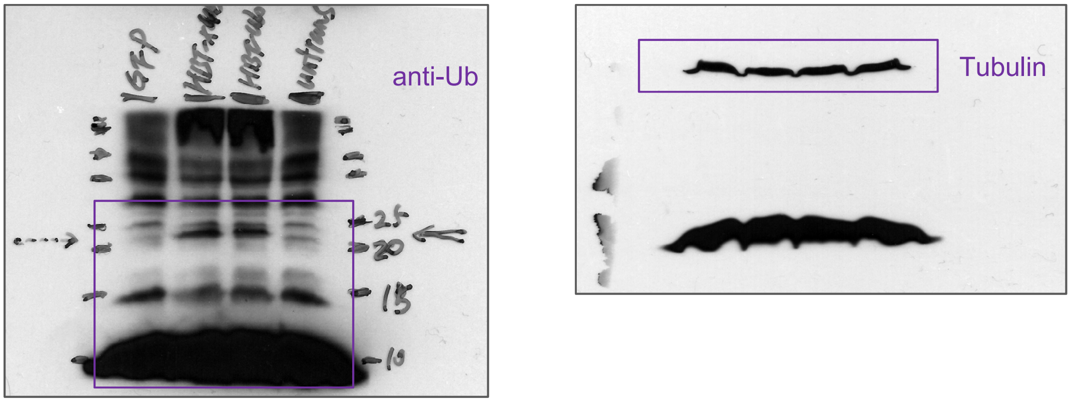

Supplementary Figure 11. Uncropped immunoblots for supplementary Fig. 2. The purple squares indicate the sections shown in supplementary Figure 2.

Supplementary Figure 12

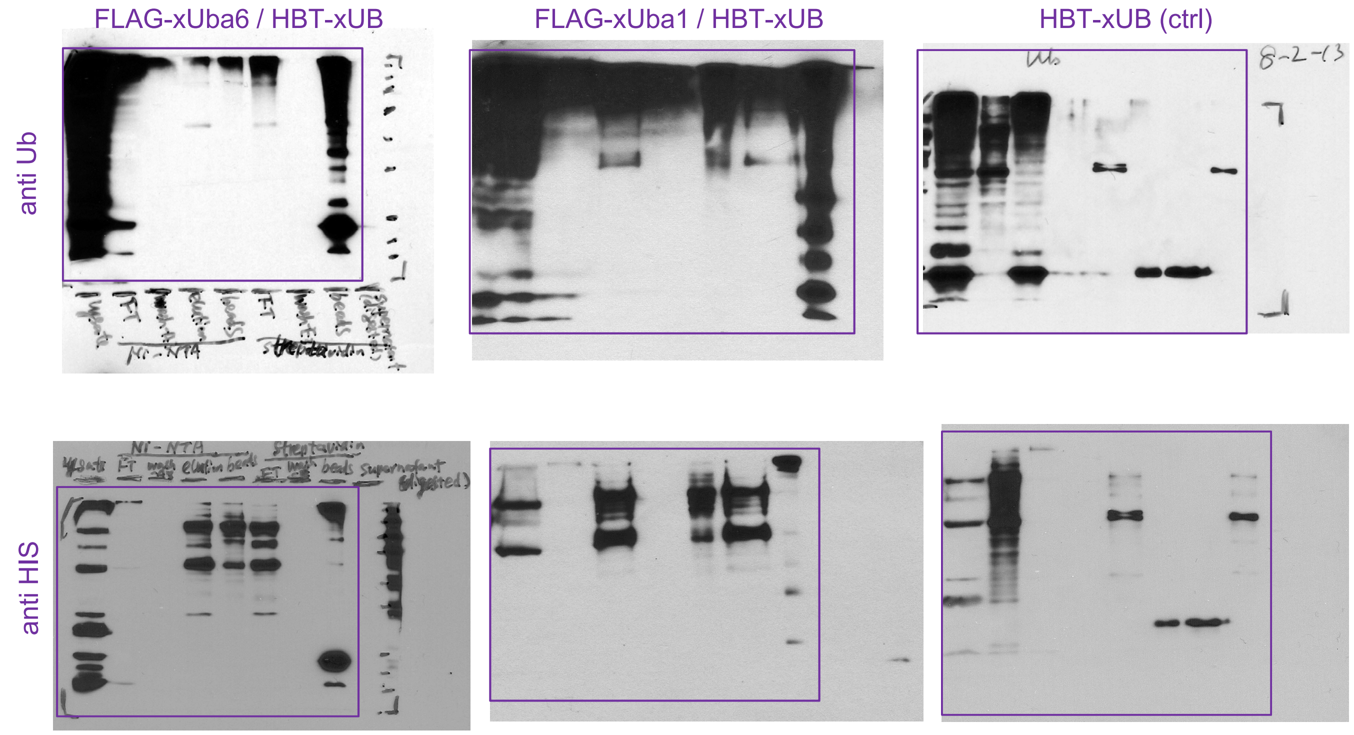

Supplementary Figure 12. Uncropped immunoblots for supplementary Fig. 3. The purple squares indicate the sections shown in supplementary Figure 3.

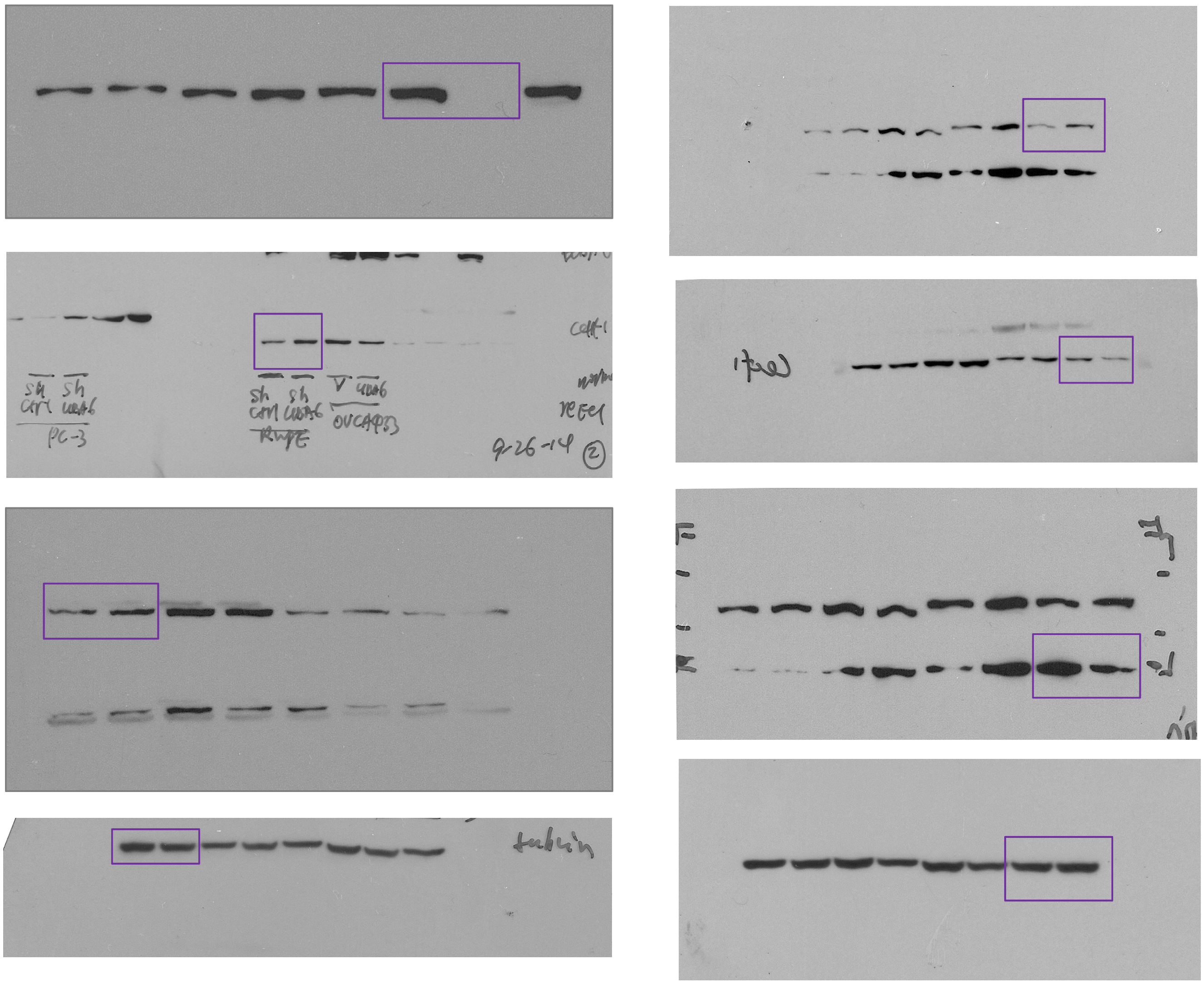

Supplementary Figure 13a. Uncropped immunoblots for supplementary Fig. 5a. The purple squares indicate the sections shown in supplementary Figure 5a.

Supplementary Figure 13b

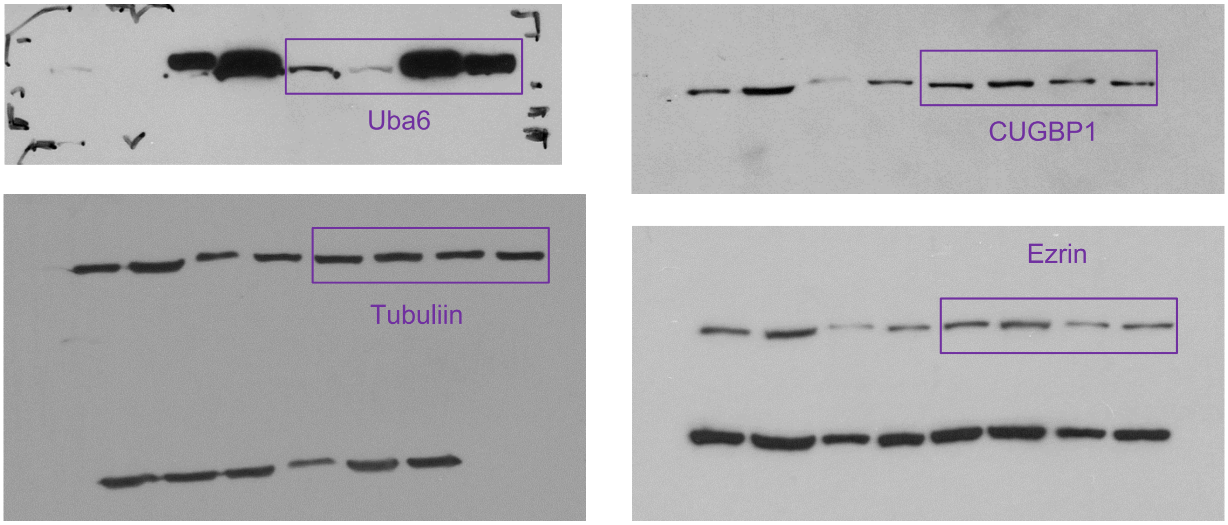

Supplementary Figure 13b. Uncropped immunoblots for supplementary Fig. 5b. The purple squares indicate the sections shown in supplementary Figure 5b.

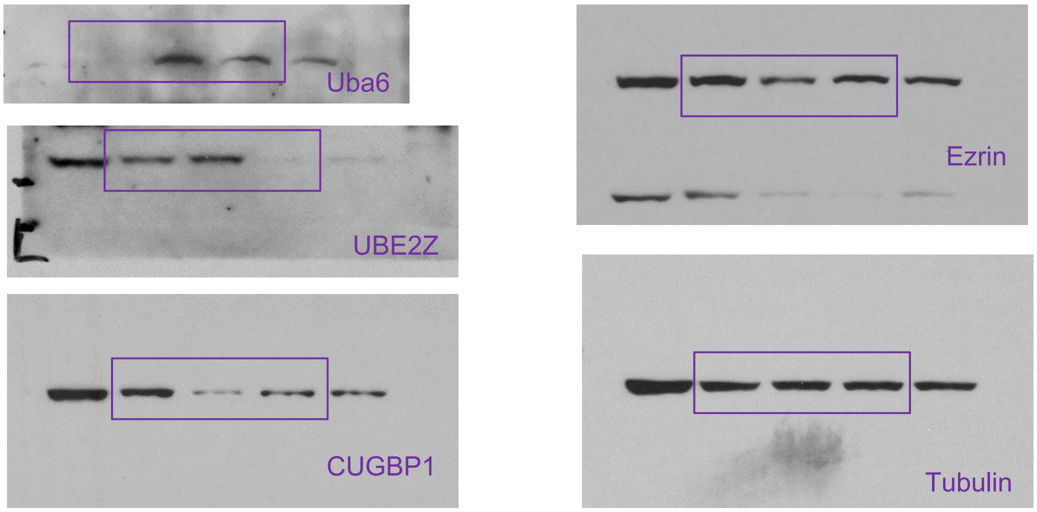

Supplementary Figure 14. Uncropped immunoblots for supplementary Fig. 6. The purple squares indicate the sections shown in supplementary Figure 6.
